# Dynamic functional connectivity and its anatomical substrate reveal treatment outcome in first-episode drug-naïve schizophrenia

**DOI:** 10.1038/s41398-021-01398-4

**Published:** 2021-05-12

**Authors:** Zhe Zhang, Kaiming Zhuo, Qiang Xiang, Yi Sun, John Suckling, Jinhong Wang, Dengtang Liu, Yu Sun

**Affiliations:** 1 Key Laboratory for Biomedical Engineering of Ministry of Education, Department of Biomedical Engineering, Zhejiang University, Zhejiang, China; 2 First-episode Schizophrenia and Early Psychosis Program, Division of Psychotic Disorders, Shanghai Mental Health Center, Shanghai Jiao Tong University School of Medicine, Shanghai, China; 3 Department of Neurology, Sir Run Run Shaw Hospital, Zhejiang University School of Medicine, Zhejiang, China; 4 Brain Mapping Unit, Department of Psychiatry, School of Clinical Medicine, Herchel Smith Building for Brain and Mind Sciences, University of Cambridge, Cambridge, UK; 5 Department of Medical Imaging, Shanghai Mental Health Center, Shanghai Jiao Tong University School of Medicine, Shanghai, China; 6 Shanghai Key Laboratory of Psychotic Disorders, Shanghai Mental Health Center, Shanghai Jiao Tong University School of Medicine, Shanghai, China; 7 Institute of Mental Health, Fudan University, Shanghai, China; 8 Zhejiang Lab, Zhejiang, China

**Keywords:** Schizophrenia, Diagnostic markers

## Abstract

Convergent evidence has suggested a significant effect of antipsychotic exposure on brain structure and function in patients with schizophrenia, yet the characteristics of favorable treatment outcome remains largely unknown. In this work, we aimed to examine how large-scale brain networks are modulated by antipsychotic treatment, and whether the longitudinal changes could track the improvements of psychopathologic scores. Thirty-four patients with first-episode drug-naïve schizophrenia and 28 matched healthy controls were recruited at baseline from Shanghai Mental Health Center. After 8 weeks of antipsychotic treatment, 24 patients were re-scanned. Through a systematical dynamic functional connectivity (dFC) analysis, we investigated the schizophrenia-related intrinsic alterations of dFC at baseline, followed by a longitudinal study to examine the influence of antipsychotic treatment on these abnormalities by comparing patients at baseline and follow-up. A structural connectivity (SC) association analysis was further carried out to investigate longitudinal anatomical changes that underpin the alterations of dFC. We found a significant symptomatic improvement-related increase in the occurrence of a dFC state characterized by stronger inter-network integration. Furthermore, symptom reduction was correlated with increased FC variability in a unique connectomic signature, particularly in the connections within the default mode network and between the auditory, cognitive control, and cerebellar network to other networks. Additionally, we observed that the SC between the superior frontal gyrus and medial prefrontal cortex was decreased after treatment, suggesting a relaxation of normal constraints on dFC. Taken together, these findings provide new evidence to extend the dysconnectivity hypothesis in schizophrenia from static to dynamic brain network. Moreover, our identified neuroimaging markers tied to the neurobiology of schizophrenia could be used as potential indicators in predicting the treatment outcome of antipsychotics.

## Introduction

Schizophrenia is a severe mental illness characterized by complex clinical symptoms, including hallucinations, delusions, and cognitive deficits^1^. The heterogeneous characteristics lead to significant challenges for efficacious treatment. In fact, more than 50% of patients received antipsychotic drugs fail to relief upon initial treatment^2,3^ despite some studies reported positive outcomes^4,5^. Continuous efforts have been made to understand the neurobiological mechanisms of antipsychotic action that lead to favorable treatment outcome. The recent advent of neuroimaging techniques have highlighted the important role of brain connectivity in response to antipsychotic treatment that could serve as potential biomarkers for treatment assessment. For instance, previous longitudinal studies in schizophrenia have revealed that the resting-state functional connectivity (FC) was increased after treatment and linked to symptoms improvement^6–8^. Other similar studies have suggested that antipsychotic medications could significantly affect the FC, but altered connectivity was not associated with the relief of psychotic symptoms^9–13^. The inconsistency of these findings may be partially due to an assumption that the functional couplings between brain regions are static, ignoring the time-varying properties of FC.

The human brain is known to be highly dynamic^14^. The recently-introduced dynamic FC (dFC) is considered to be an efficient way for uncovering specific functional integration properties that cannot be discovered through conventional static FC^15–17^. Of note, schizophrenia is beginning to be revealed as abnormalities of dFC^18–22^. Specifically, studies using graph theory-based approaches have revealed a disruption in dFC spatiotemporal efficiency^23,24^ and temporal variability^25,26^, which could serve as potential biomarkers for schizophrenia classification^27^^.^ However, only one study, to the best of our knowledge, has attempted to examine the effects of antipsychotic exposure on dFC properties of schizophrenia and showed treatment effects of risperidone on dFC in insular in first-episode drug-naïve (FEDN) schizophrenia^28^. It is still largely unknown how antipsychotic treatment might modify dynamic patterns in large-scale brain networks and whether these changes are related to symptomatic improvement longitudinally.

It is widely accepted that brain function is partially constrained by its anatomical substrates^29,30^, with ample evidence of spatial correspondence between structural connectivity (SC) and FC^31,32^. Thus, a comprehensive understanding of the effects of treatment on dFC should also consider SC as a scaffold for coordinated neural activity. For patients with schizophrenia, overlapping alterations of structure and function have been repeatedly reported^33–36^. Additionally, the relationship of SC–FC within specific networks appears to be absent in patients with schizophrenia^37^. Together, these findings suggest that disruptions of SC–FC coupling are implicated in the pathophysiology of schizophrenia. To date, no prior study has applied a combination analysis of SC and dFC to explore the effects of antipsychotic treatment on large-scale brain networks of schizophrenia.

The primary aim of this study is to investigate medication-induced dFC changes in large-scale brain networks in FEDN schizophrenia related to the relief of psychotic symptoms. A systematic analysis was performed to explore the dFC differences at baseline between patients and controls, followed by a longitudinal study to determine how altered dFC patterns change in response to antipsychotic treatment by comparing patients at baseline and follow-up. Furthermore, we performed an SC association analysis to examine the longitudinal changes in anatomical constraints that support altered dFC in baseline patients, as well as longitudinal changes of its coupling with dFC. In particular, the SC with altered FC variability in baseline patients will be explored, so that we can focus the longitudinal changes of SC–dFC coupling on individual connection. Based upon the previous evidence supporting aberrant brain connectivity in schizophrenia, we hypothesized that: (1) the altered dFC in baseline patients is modified by treatment and tracks the improvement of psychopathologic scores; and (2) SC constrains altered dFC and their relationships at baseline in patients is modulated by antipsychotic treatment.

## Materials and methods

### Participants

Forty-six patients with FEDN schizophrenia and 32 matched healthy volunteers were recruited from the Shanghai Mental Health Center (SMHC) and local community. All the patients fulfilled the DSM-5 diagnostic criteria for schizophrenia, and all of them did not have any form of antipsychotic treatment prior to the initial scan. The detailed inclusion and exclusion criteria are described in Supplementary materials and Fig. S1. This study was approved by the Institutional Review Board of SHMC.

### Medication and clinical assessments

The patients with FEDN schizophrenia were treated with second-generation antipsychotic (SGAs) for 8 weeks following the guidelines according to refs. ^38,39^. Detailed information of medication and dosage for each patient is available in Table S1. The psychopathological symptoms of each patient were measured using the PANSS^40^.

### Image acquisition and preprocessing

All MRI data including T1-weighted, resting-state fMRI, and diffusion-weighted imaging (DWI) were acquired on a 3.0T Siemens Tim Verio scanner (Siemens, Germany) at SMHC. Patients were scanned both at baseline and 8 weeks follow-up, and healthy controls were scanned only at baseline. Participants were instructed to stay awake with their eyes closed, to remain still, and not to think systematically during the scan. Functional images were preprocessed using the DPARSF toolbox^41^, including removal of the 5 initial volumes, slice-timing, realignment, normalization, and spatial smoothing. Diffusion images were preprocessed using the PANDA toolbox^42^, which included estimating the brain mask, cropping the raw images, correcting for the eddy-current effect, and calculating diffusion metrics. Of note, to account for artifact of head motion, any data affected by head motion (maximal motion between volumes in each direction is greater than 1.5 mm, and rotation in each axis is greater than 1.5°) were discarded. We also calculated the mean frame-wise displacement (FD) of each participant on the basis of realignment parameters and the participants with FD more than 0.2 mm were excluded. After head motion exclusion, 34 patients and 28 controls at baseline and 24 patients at follow-up were included in the subsequent analysis. Greater details of data preprocessing and criteria for significant head motion are provided in Supplementary methods. The analysis framework of this study is shown in Fig. S2.

### Group independent component analysis

To identify the intrinsic connectivity networks (ICNs) and their corresponding time courses, we parcellated the fMRI data using a standard pipeline for spatial group independent component analysis (ICA)^43,44^, including principal component analysis (PCA) to reduce subject-specific data, informax algorithm analysis to further reduce the concatenated subject-reduced data into independent components (ICs)^45^, back reconstruction from group spatial maps to subject-specific spatial maps and estimate the corresponding time courses via group ICA approach. Of note, the global signal regression was performed as the standard PCA processing step prior to ICA implemented by GIFT toolbox. The ICA process resulted in 36 ICNs that were categorized into 7 resting-state networks (RSNs) including sub-cortical (SUC; 2 ICNs), auditory (AUD; 2 ICNs), visual (VIS; 5 ICNs), somatomotor (SM; 9 ICNs), cognitive control (CC; 9 ICNs), default mode (DM; 7 ICNs), and cerebellar (CB; 2 ICNs) networks. The activation information spatial maps of these ICNs are presented in Table S2 and Fig. S3. Additionally post-processing steps were performed on the time courses of the 36 ICNs to remove remaining noise sources, including detrending linear, quadratic and cubic signals, conducting regressions of the 6 realignment parameters and their temporal derivatives, despiking detected outliers, and low-pass filtering with a cutoff frequency of 0.15 Hz. See Supplementary materials for more details.

### Dynamic functional connectivity estimation

A previously validated sliding window approach was used here to estimate dFC. Here, the time courses of ICNs were divided into a window with 22 TRs length and 1 TR increment-step, resulting in a total of 153 windows across the entire scan for each participant. Of note, we used the window length of 22 TRs since it has been demonstrated to provide a good cutoff between the quality of the correlation matrix estimation and the ability of to detect functional dynamics^16^. A 36 × 36 covariance matrix within each window was calculated to construct FC networks^46^, wherein each ICN was defined as a node and the connectivity between two ICNs was defined as an edge. An L1 norm in the graphic LASSO framework with 100 repetitions^47^ was further conducted to promote sparsity in estimation. The obtained FC matrices were then converted to z-scores using Fisher’s z-transformation and residualized with nuisance variables. The resulting 153 matrices for each participant represent the dynamic changes of FC networks over time. Since previous studies have suggested that dynamic connectivity patterns could be captured stably in the range of 30–60 s^16,48^, we verified the findings by testing other window sizes (18–26 TRs).

### Dynamic functional connectivity analysis

We then performed a systematical analysis to explore the treatment-related reorganization of dFC at multiple topological scales, ranging from the whole network to individual connectivity edges, with focus on the temporal properties of dFC states, dynamic network efficiency, and temporal variability of FC.

#### Temporal properties of dFC states

A k-means clustering analysis was initially performed on the dFC matrices of all participants to estimate reoccurring dFC states. The similarity between matrices and cluster centroids was measured using the L1 distance function^49^. The optimal number of clusters = 5 was estimated by using the elbow criterion^18^. The obtained group centroids were used as the initial centroids to cluster each participant’s windowed FC matrices. Of note, a state was considered as a reliable state when it covered at least 10 windows. Additionally, we conducted a k-means clustering analysis with other numbers of clusters (k = 4, 6) to verify the findings in this study. The temporal properties of dynamic states were then evaluated using a measure of state occurrence rate (the proportion of time spent in each state). A Mann–Whitney *U*-test was used to test the significance of a state occurrence between baseline patient and control groups, and a paired t-test was conducted between patients at baseline and follow-up. A FDR-corrected *P* < 0.05 was considered as significant.

#### Dynamic network efficiency

A network efficiency approach was then applied to examine the temporal variability of the topological organization of large-scale brain networks at both global and local level. Efficiency was calculated at a several sparsity thresholds from 0.1 to 0.35 according to ref. ^43^. The area under the curve (AUC) within the defined threshold range for each measure of efficiency was estimated. The variance on the AUC changes across 153 windows was calculated to examine dynamic topology properties of the FC networks^24^. The graph theory analyses were performed with GRETNA software (http://www.nitrc.org/projects/gretna). A Wilcoxon rank test was used to test the group difference in dynamic network efficiency (*P* < 0.05, FDR-corrected).

#### Temporal variability of FC

The temporal variability of FC was measured by calculating the FC variance in each pair of ICNs across all windows, which plays a key role in dynamically integrating and coordinating neural systems^50,51^. Then the Network Based Statistics (NBS)^52^ was used to identify whether there was a group difference in connectome-based FC variability by comparing the baseline patient and control groups. Different from traditional statistical analysis, the NBS handles the multiple comparisons problem of the multiple edges in connectomic data by evaluating the null hypothesis at the level of inter-connected sub-networks rather than individual connections^52^. NBS inference was by permutation testing using unpaired *t* tests with 5000 permutations. A test statistic was then computed for each connection, and a threshold of *t* = 2.708 was applied to identify a set of supra-threshold connections that showed significant differences in FC variability between baseline patient and control groups (significance at *P*<0.05, FDR-corrected). To examine the longitudinal changes of FC variability after treatment, we used a paired t-test to test the group difference between patients at baseline and follow-up with permutation inference (*P* < 0.05, FDR-corrected).

### SC–dFC association analysis

To observe how the anatomical constraints of altered dFC change in response to treatment, we used DWI to inform the SC between pairs of ICNs that showed altered FC variability in baseline patients. We transformed the ICNs in the MNI space to the native DWI space of each participant. First, the ICBM 152 template was registered to the b0 image of each participant in native DWI space using linear and nonlinear registration in FSL. Then, atlases from ICNs were transformed to each participant’s native space with nearest-neighbor interpolation. White matter tracts were traced using a whole-brain deterministic tractography approach, which was performed in the native space for each participant using the fiber assessment by continuous tracking (FACT) algorithm. Here, fiber tracking was terminated when the angle between two consecutive orientations was >45º or when the FA value was <0.2. In line with previous studies^36^, the number of diffusion streamlines connecting pairs of regions was used as a measure of SC weights, which has been demonstrated as effective in detecting anatomical abnormalities both in first-episode and chronic schizophrenia patients^53,54^. The strength of coupling between SC and dFC was examined by computing the level of Spearman’s correlation between the weights of existing SC and its dFC counterparts. This correlation is referred to as the level of SC–dFC coupling (*P* < 0.05). Detailed information of SC estimation is provided in Supplementary methods.

### Clinical and psychological data analysis

Group differences in demographics were explored using a two-sample *t*-test (age and education) and a chi-square test (gender). Differences in PANSS scores between patients at baseline and follow-up were examined using a paired *t*-test. Furthermore, we carried out Spearman’s correlation analyses to investigate the relationships between alterations in brain measures (dFC and SC) and PANSS scores as well as between their longitudinal changes in schizophrenia patients, controlling for age, gender, mean frame-wise displacement, and daily dose of antipsychotic medication. Statistical analyses were performed using SPSS 24.0 (IBM Corporation, Armonk, NY, USA) and statistical significance was set at *P* < 0.05 with FDR-correction.

## Results

### Demographic and clinical characteristics

Healthy controls and patients at baseline did not differ in age, gender, and education (all tests *P* > 0.05). The mean duration since onset of psychiatric symptoms for baseline patients was 6.7 months (SD = 11.9). After 8 weeks of antipsychotic treatment, we found a significant reduction on PANSS total score, positive symptoms, negative symptoms, and general symptoms (*P* < 0.0001). Detailed demographic and clinical characteristics of the included participants are shown in Table 1.

**Table 1:** Demographics and clinical characteristics from study groups.

	SZ_b (*n* = 34)	SZ_p (*n* = 24)	HC (*n* = 28)	SZ_b verses HC (*P*-value)	SZ_bp verses SZ_p (*P*-value)
Age (y)	27.1 ± 6.1	27.4 ± 5.7	27.1 ± 4.5	0.99^c^	\
Gender (f)	17	12	15	0.78^d^	\
Handedness (l/r)	0/34	0/24	0/28	\	\
Education (y)	12.9 ± 3.2	13.2 ± 3.2	14.4 ± 2.2	0.06^c^	\
^a^DUP (m)	6.7 ± 11.9	\	\	\	\
^b^Antipsychotic dose(in CPZE, mg)	\	339.6 ± 137.5	\	\	\
PANSS total score	74.0 ± 17.8	48.3 ± 14.0	\	\	<0.0001^e^
positive symptoms	20.3 ± 5.3	11.0 ± 3.6	\	\	<0.0001^e^
negative symptoms	16.6 ± 6.1	12.3 ± 4.8	\	\	<0.0001^e^
general symptoms	37.1 ± 9.4	25.0 6.7	\	\	<0.0001^e^

Values are mean ± SD or N.

*y* year, *f* female, *m* month, *l* left, *r* right, *DUP* duration of untreated psychosis.

^a^DUP of each FEDN schizophrenia patient was determined by psychiatrists from the disease onset until the first clinical examination.

^b^The daily antipsychotic dose converted to chlorpromazine equivalents (CPZE).

^c^Statistical differences were obtained using two-sample *t*-test.

^d^Statistical differences were obtained using Chi-square test.

^e^Statistical differences were obtained using paired *t*-test.

### Occurrences of dFC patterns

**Fig. 1:**
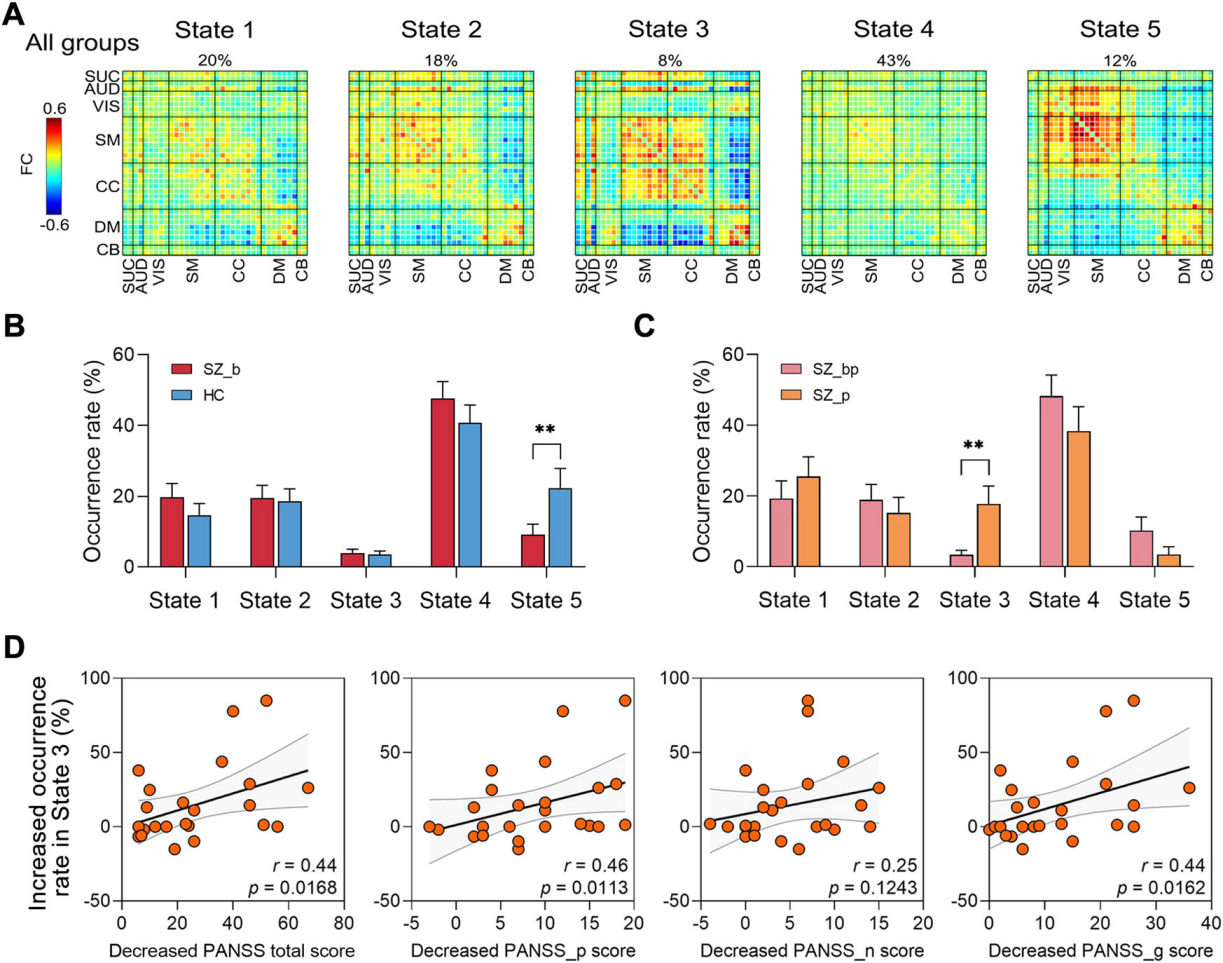
Dynamic functional connectivity patterns and association with psychosis symptoms. **A** Five discrete dynamic functional connectivity patterns across all groups. The percentage of occurrences is listed above each cluster centroid. The color bar represents *z* values of functional connectivity (FC). **B** Differences in state occurrences between baseline schizophrenia patient (SZ_b) and healthy control (HC) groups. **C** Group differences in state occurrences between post-treatment (SZ_p) and paired baseline patients (SZ_bp). **D** Associations between changed occurrences of the State 3 and improved psychosis symptoms after treatment. **P < 0.01, FDR-corrected.

The cluster centroid of 5 dFC states across participants is shown in Fig. 1. Supplementary Fig. S4 shows group-specific cluster centroids for each state. Compared with controls, baseline patients showed a lower occurrence rate in State 5 (*P* = 0.0098, FDR-corrected), which exhibited strongly positive couplings between AUD and VIS, and SM networks as well as between VIS and SM networks, representing a high sensory-network interaction state. We then investigated the longitudinal changes of state occurrence in response to antipsychotic treatment and observed that the occurrence rate in State 3 was significantly increased in patients after treatment compared with that at baseline (*P* = 0.0058). State 3 exhibited strongly positive couplings among SUC, AUD, SM, and CC, as well as negative DM couplings with SUC, AUD, SM, and CC, representing a strong inter-network integration state. Spearman’ correlation analysis further showed that the increased occurrence rate in State 3 was significantly related with decreased PANSS score after treatment. Moreover, we found that these findings were independent of different settings of sliding window (Figs. S5, S6) and clusters (Figs. S7, S8), and global signal (Fig. S9), indicating a medication-induced intrinsic reorganization of dFC that were associated with relief of psychotic symptoms after treatment.

### Temporal variability of dFC

**Fig. 2:**
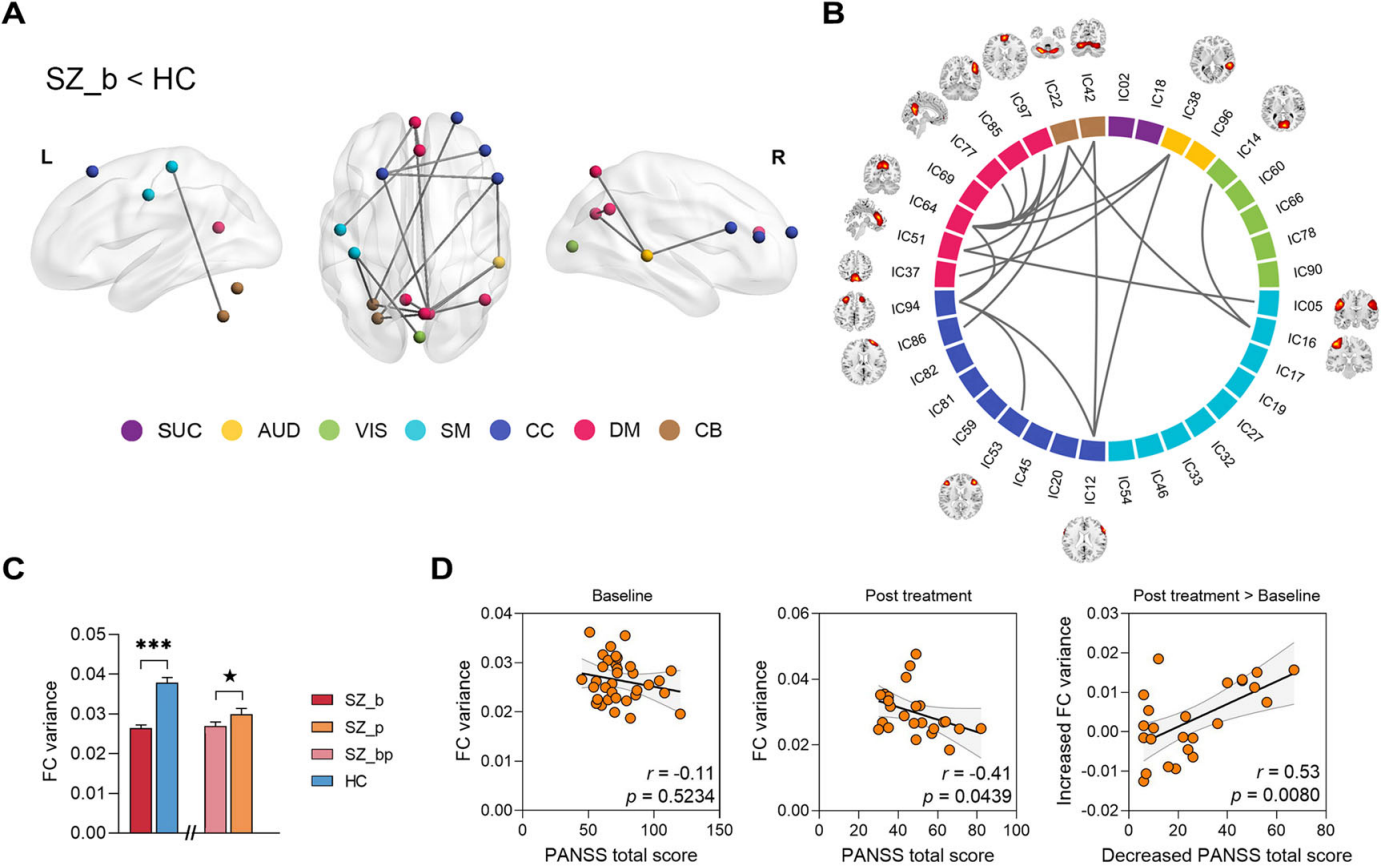
The connectomic signature and association with psychosis symptoms. **A**, **B** The functional connectomic signature was identified by using the NBS at baseline (SZ_b < HC). **C** Group differences in FC variability of this signature. **D** Associations between FC variability and psychosis symptoms. ***P < 0.001, ^★^*P* = 0.058, FDR-corrected.

We investigated the dynamic network efficiency in time-varying functional brain networks and found insignificant difference between both groups at baseline (Fig. S10). We then explored the schizophrenia-related alterations of FC variability and revealed a pre-treatment connectomic signature that showed a significant difference in FC variance between both groups at baseline (SZ_b < HC; *P* = 0.016, FEW-corrected). This signature was characterized by lower FC variability within DM and CC as well as between multiple other RSNs (i.e., DM and AUD, SM, CC, CB; CC and AUD, CB; SM and VIS, CB) (Fig. 2). Moreover, we observed that the FC variability of this signature was increased in patients after treatment compared with that at baseline, although the group difference showed a marginally statistical significance (*P* = 0.058).

**Fig. 3:**
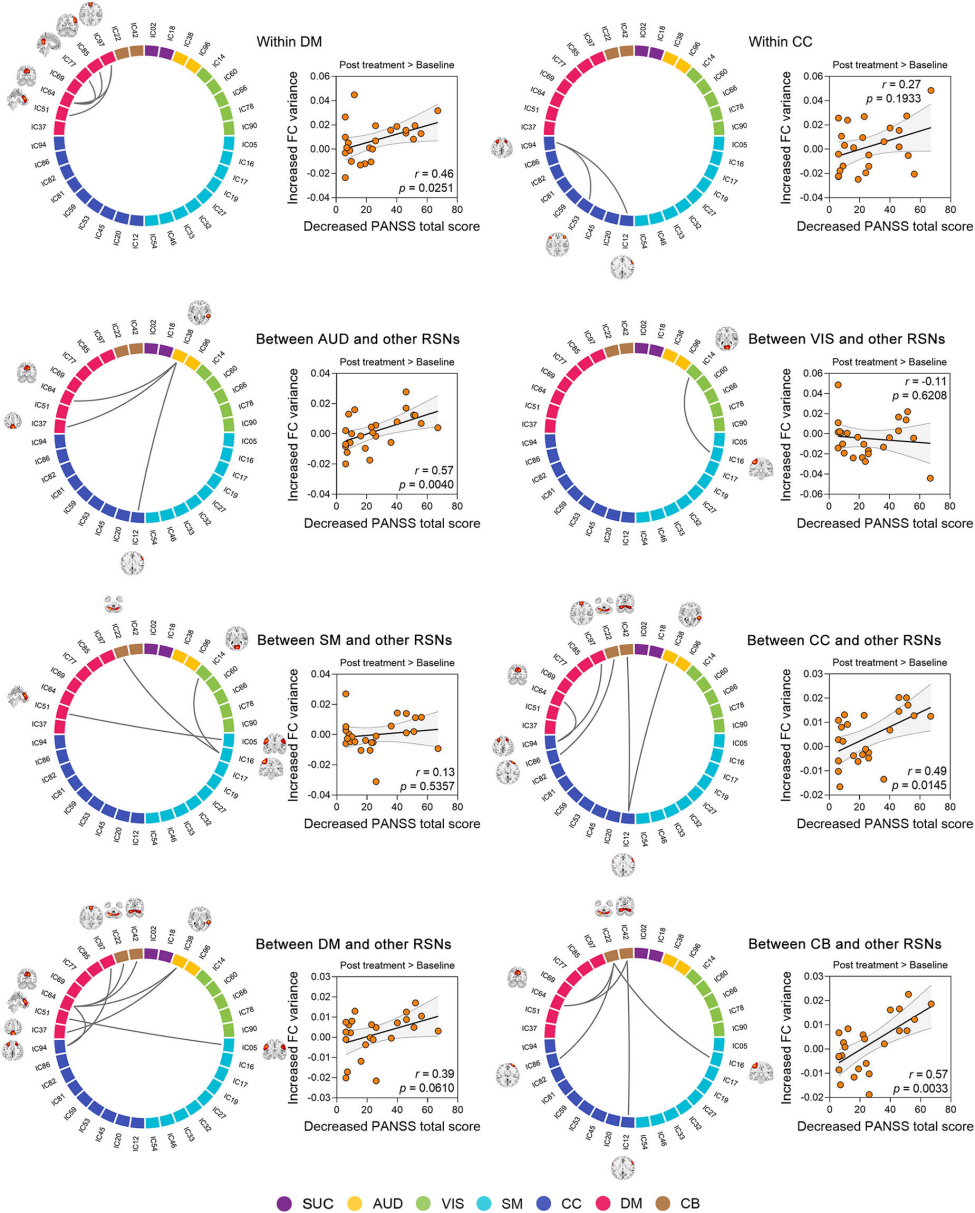
Associations between altered FC variability of intra- and inter-RSNs and relief of psychosis symptoms. Significance, *P* < 0.05, FDR-corrected.

We further evaluated the association between FC variability and clinical measures in patients. There was no significant correlation at baseline (*r* = −0.11, *P* = 0.5234), but a significantly negative correlation at follow-up (*r* = −0.41, *P* = 0.0439). Importantly, we observed that increased FC variability was significantly correlated with decreased PANSS total score after treatment (*r* = 0.53, *P* = 0.0080). Further analysis indicated that the significant correlations (*P* < 0.05) involved the intro-network connections of DM, as well as the inter-network connections between AUD, CC, and CB to other RSNs (Fig. 3). These analyses suggest that relief of psychotic symptoms in patients after treatment is associated with increased FC variability within and between specific brain networks.

### Association between dFC and SC

**Fig. 4:**
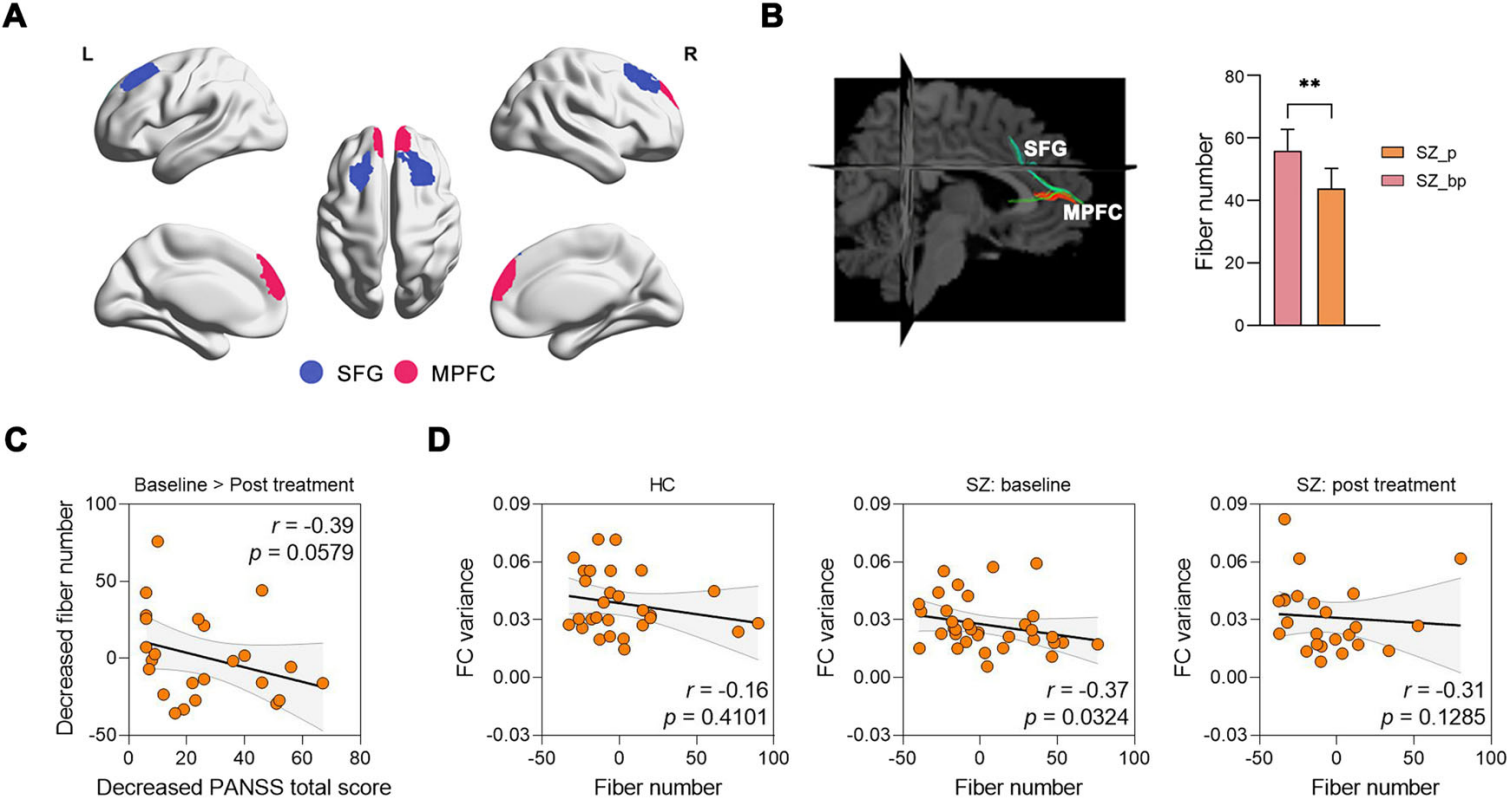
Structural connectivity association analyses. **A** Spatial maps of two components (SFG and MPFC). **B** Differences in number of diffusion streamlines in patients between baseline (SZ_p) and follow-up (SZ_bp). **C** Decreased number of streamlines between the SFG and MPFC were marginally negatively correlated with decreased PANSS scores in patients after treatment. **D** Relationship between FC variability and number of streamlines in each groups. **P < 0.01. SFG = superior frontal gyrus; MPFC = medial prefrontal cortex.

To investigate the longitudinal changes in SC that support altered dFC in baseline patients, we tracked the number of DWI streamlines connecting pairs of ICNs identified as connectomic signatures as having lower FC variability. We found that the number of reconstructed streamlines between IC94 (SFG) and IC97 (MPFC) was significantly decreased in patients after treatment compared with that baseline (*P* = 0.0023) (Fig. 4). Additionally, decreased fiber numbers were negatively correlated with decreased PANSS scores after treatment (*r* = −0.39, *P* = 0.0579). We further observed the relationships in the SFG-MPFC connection between FC variability and number of fiber streamlines for each group. A significant association between FC variability and number of fiber streamlines was found at baseline in patients (*r* = −0.37, *P* = 0.0324), while this relationship was not present in controls (*r* = −0.16, *P* = 0.4101) or treated patients (*r* = −0.31, *P* = 0.1285), suggesting a decoupling of SC and dFC for schizophrenia patients in response to antipsychotic treatment.

## Discussion

In this study, we performed a systematical dFC analysis to investigate how antipsychotic medication modifies large-scale brain networks in FEDN schizophrenia and how these alterations relate to changes in symptoms. The present results establish a significant relationship between brain networks and clinical relief. Specifically, the longitudinal changes of dFC state occurrence and FC variability emerged as key indicators of symptomatic improvement in schizophrenia. Moreover, SC association analyses indicate that the anatomical constraints to support dFC and their association were modified longitudinally by antipsychotic treatment. Thus, the present work provides the novel evidence for neurobiological mechanisms of antipsychotic treatment that lead to symptomatic improvement, and underscore the potential of dynamic functional brain networks in searching prognostic markers and predicting treatment response of schizophrenia.

### Occurrence of dFC patterns changes longitudinally and tracks symptomatic improvement

Our results demonstrated significant group differences in the occurrence of dFC states. That the baseline patients spent much less time than controls in State 5 is consistent with prior findings in chronic schizophrenia^18,20,22^. State 5 was characterized by strong interactions between sensory networks, and it represents a state of low alertness or drowsiness in the human brain^16^. The lower occurrence of this state may reflect an excessive level of arousal or an inability to be in relaxation that may lead to diverse symptoms of schizophrenia^22^. It can be supported by the relationships established in our study, where the lower occurrence in State 5 was linked to more severe clinical symptoms (Fig. S11). More importantly, we found that the occurrence of State 3 in patients after treatment was higher than baseline. This finding is partly consistent with a previous work that the dwell time of dFC states was changed in patients with chronic schizophrenia after 6 weeks of risperidone treatment^11^. Specifically, state 3 was characterized by stronger positive couplings among multiple networks (i.e. SC, AUD, SM and CC), as well as negative correlations between DM and these networks. The increased occurrence of this state may reflect a reinforced global integration of the brain networks in patients after treatment and thus contribute to the improvement of psychosis symptoms. Our results could also be interpreted as a potential compensatory mechanism of dFC states in response to antipsychotic treatment, where antipsychotic drugs did not normalize the occurrence of a strong sensory-network interaction state, but instead enhanced the expression of a strong inter-network integration state. Future studies are required to clarify the relevance of these dFC states, particularly State 3, with brain cognitive states, as well as their neurophysiological mechanism in response to antipsychotic treatment.

### FC variability changes longitudinally and tracks symptomatic improvement

Our findings of a baseline deficit in dFC variability in patients are consistent with a report of reduced FC dynamism in clinical high-risk individuals for schizophrenia^55^. It has been suggested that a higher FC variability may reflect a greater complexity and greater capacity for information processing^26,56,57^. Thus, the lower FC variability we observed may indicate decreased ability for information processing in patients. Moreover, we found that FC variability of this connectomic signature was increased in patients with greater symptomatic reduction after treatment, suggesting a medication-induced normalization. Indeed, the findings of time-invariant FC studies have reported that successful treatment is often linked to the normalization of regional FC^9,13^. It is therefore intriguing to assume that the increases in FC variability caused by antipsychotic medications may modulate the corresponding spatiotemporal changes on the psychological level which, in turn, improve psychosis symptoms, corresponding to what has been described as “Spatiotemporal Psychopathology”^58,59^. Further results demonstrated that the symptomatic improvement-related FC variability in this signature was mainly involved with intra-network connections of DM and inter-network connections of AUD, CC, and CB, which are well known to be implicated in schizophrenia. Specifically, dysfunction in the DM may lead to the disturbances of self-referential processing in patients^60^. Convergent evidence has linked symptomatic improvement to FC changes within the DM^9,13,61^. Consistently, our increased FC variability within the DM suggest that self-related thought plays a pivotal role in recovery from psychosis symptoms. Additionally, dysconnectivity of the primary sensory networks (e.g., AUD and CB) with other high-order cognitive networks (e.g., CC) is considered to be related to symptoms such as hallucinations and emotional dysregulation^62–65^, and abnormal CC connectivity to other networks might lead to various executive control deficits in patients with schizophrenia^66,67^. Overall, our findings, therefore, provide solid evidence supporting that symptomatic reduction in response to antipsychotic treatment is associated with normalization of FC variability in multiple large-scale networks implicated in the pathophysiology of schizophrenia.

### SC–dFC coupling changes longitudinally

A particularly important objective of our study was to explore whether the structure underpinnings of altered FC variability in baseline patients and their relationship can be modulated by antipsychotic treatment. It has been suggested that antipsychotic medications have an effect on myelin content, thus changing whiter matter (WM) microstructure. Studies using DTI have frequently revealed WM tract changes in relation to 6–12 weeks of antipsychotic exposure, ranging from white matter integrity to network topologic organization^54,68–70^. Our results add to previous findings, suggesting that the SC between the SFG and MPFC can be modulated by pharmacological intervention, especially during the early phase of treatment. However, it is not possible to entirely exclude the influences of underlying progression of illness. We also observed that symptom reduction was anti-correlated with decreased SC between the SFG and MPFC, suggesting that antipsychotic drugs might attenuate clinical features of schizophrenia by delaying the reduction of WM tracts. Our results can be supported by previous findings that antipsychotic-treated patients exhibited much less reduction of fractional anisotropy than never-treated patients in brain structure^71^. Moreover, we observed that the FC variability between the SFG and MPFC was negatively correlated with its SC counterparts in baseline patients, but this relationship was lost after treatment, suggesting that antipsychotic treatment may induce a decoupling of SC–dFC on this connection. Contemporary theories have suggested that aberrant relationships of SC and (static) FC could lead to deficits in executive function as well as the emergence of neuropsychiatric disorders such as schizophrenia^36,72–74^. In this study, we extend these findings by showing a tightly coupled SC and dFC at baseline in patients, which reflects a less dynamic or more stringent functional communication between the SFG and MPFC, may be directly constrained by its underlying white matter structure. Echoing the triple-network hypothesis of schizophrenia^75,76^, the decoupling after treatment suggests an increased functional flexibility and dynamic recruitment to balance internally generated thoughts and externally stimulated executive processing^77,78^, and thus contribute to the relief of psychotic symptoms in schizophrenia patients.

### Clinical implications

Our findings may contribute to the future therapy for schizophrenia in the following two aspects. First, our study sheds light on the neural pathophysiology underlying therapeutic effects of antipsychotic treatment. Although attempts have been made to determine neuroimaging-based biomarkers that relate to treatment response, the findings are inconsistent^6–12^. Our study, from the perspective of large-scale brain networks, provides a new avenue for exploring potential markers of treatment that contribute to symptom reduction. Second, our study provides important information in the search for clinically useful prognostic markers of schizophrenia. Recently, many researchers have examined whether brain connectivity can be used to predict response to antipsychotic treatment^54,79–82^. In our study, we used baseline dFC features to predict symptomatic improvement after treatment. Our results indicate that the dFC features can significantly improve prediction performance compared to models using demographic and clinical measures alone. Specifically, we obtained the cross-validated accuracy of a model without dynamic features/with dynamic features = 0.40/0.55, *P* = 0.0552/0.0052 (Fig. S12). Given the limited sample sizes in our study, however, this high prediction performance should be verified in further longitudinal research.

### Limitations

Our study was subject to a few limitations that deserve mention. First, to investigate the changes caused by direct antipsychotic treatment rather than a potential effect of the disease itself, we opted for a modest homogeneous cohort of FEDN schizophrenia instead of a large heterogeneous cohort. Our patients are older than the patients included in previous studies^82,83^. Future study with a larger and younger homogeneous cohort is needed to validate our findings. Second, all patients were treated with the SGA medication. However, previous findings have suggested that different antipsychotic agents vary in their potency and effects on brain structure and function^3^. It would be important for a further study to determine whether the changes observed here were general or specific across different types of antipsychotics. Third, although our study has focused on the structural underpinnings of a connectomic signature with lower FC variability, it has been suggested that medication-induced SC changes can be preserved between brain regions that show normal functional coupling. That means the alterations of SC–dFC coupling after treatment can be found even on the connections outside the connectomic signature. Therefore, future studies in schizophrenia should measure the treatment-induced changes in whole-brain SC–dFC coupling.

In conclusion, our study investigated the modification of large-scale brain networks caused by antipsychotic medications in FEDN schizophrenia. We have shown, for the first time, that longitudinal changes of occurrence of dFC states and FC variability can track symptomatic improvement in response to antipsychotic treatment. Furthermore, the SC constraints of altered dFC, as well as their relationships, are longitudinally altered by medications. Overall, these findings demonstrate that antipsychotic treatment can ease psychotic symptoms by modulating large-scale brain networks, which opens up new avenues to develop a target for novel therapeutic interventions and to identify an indicator of long-term prognosis.

## Supplementary information

Supplementary materials

## Data Availability

The data that support the findings of this study are available from the corresponding author, upon reasonable request.
