# Supplementary material for: Dynamic functional connectivity and its anatomical substrate reveal treatment outcome in first-episode drug-naïve schizophrenia
Source: Transl Psychiatry. 2021 May 12;11:282. doi: 10.1038/s41398-021-01398-4 (PMC8115129; doi:10.1038/s41398-021-01398-4)
Supplement: Supplementary file 1 — Supplementary materials [file 41398_2021_1398_MOESM1_ESM.docx]

Supplementary Information for

Dynamic functional connectivity and its anatomical substrate reveal treatment outcome in first-episode drug-naïve schizophrenia

Zhe Zhang, Kaiming Zhuo, Qiang Xiang, Yi Sun, John Suckling, Jinhong Wang, Dengtang Liu, Yu Sun

Corresponding Author:

Yu SUN, Email: [yusun@zju.edu.cn](mailto:yusun@zju.edu.cn);

Dengtang Liu, Email: [liudengtang@smhc.org.cn](mailto:liudengtang@smhc.org.cn)

**Contents**

[Supplementary Methods 3](#_Toc93)

[Participants 3](#_Toc12225)

[Overview of the longitudinal connectivity analyses 3](#_Toc11754)

[Data acquisition 4](#_Toc30182)

[Functional image preprocessing 5](#_Toc24638)

[Diffusion image preprocessing 5](#_Toc32064)

[Group independent component analysis 6](#_Toc26950)

[Structural connectivity estimation 7](#_Toc5762)

[Validation analysis 7](#_Toc19351)

[Supplementary Results and Discussion 8](#_Toc1002)

[Group-specific cluster centroids for each state 8](#_Toc27249)

[Occurrences of dynamic functional connectivity patterns in different window sizes 8](#_Toc1483)

[Occurrences of dynamic functional connectivity patterns in different cluster number 8](#_Toc3746)

[Occurrences of dynamic functional connectivity patterns without GSR 9](#_Toc19548)

[Dynamic network efficiency 9](#_Toc3998)

[Supplementary Tables and Figures 11](#_Toc243)

[Table S1. The information of medication and dosage for each patient. 11](#_Toc10833)

[Table S2. Peak activation information of the 36 ICNs 12](#_Toc27155)

[Fig. S1 Flow chart showing the inclusion of patients for this longitudinal study. 15](#_Toc7019)

[Fig. S2 Analysis flowchart to study brain network connectivity in first-episode schizophrenia. 16](#_Toc2296)

[Fig. S3 Composite maps of the 36 identified ICNs. 17](#_Toc320)

[Fig. S4 Group-specific centroid matrices. 18](#_Toc17548)

[Fig. S5 Dynamic functional connectivity patterns with 18 TRs. 19](#_Toc26991)

[Fig. S6 Dynamic functional connectivity patterns with 26 TRs. 20](#_Toc5345)

[Fig. S7 Dynamic functional connectivity patterns with 4 clusters. 21](#_Toc20713)

[Fig. S8 Dynamic functional connectivity patterns with 6 clusters. 22](#_Toc31657)

[Fig. S9 Dynamic functional connectivity patterns without global signal regression. 23](#_Toc20754)

[Fig. S10 Dynamic network efficiency and its association with clinical symptoms. 24](#_Toc14661)

[Fig. S11 Associations between the occurrence of State 5 and PANSS scores in baseline patients. 25](#_Toc19223)

[Fig. S12 Prediction of symptom reduction using clinical and brain network measure features. 26](#_Toc27479)

[Supplementary References 27](#_Toc10914)

# Supplementary Methods

## Participants

Forty six patients with drug-naïve, first-episode schizophrenia were recruited from outpatient at Shanghai Mental Health Center. All of them fulfilled the Diagnostic and Statistical Manual of Mental Disorders, Fifth Edition (DSM-5) (American Psychiatric Association, 2013) diagnostic criteria for schizophrenia or schizophreniform disorder, and the diagnosis was confirmed by a research psychiatrist (Zhuo, K.) using the Mini International Neuropsychiatric Interview (MINI 7.0) ^1^. The patients diagnosed with schizophreniform disorder at the time of study enrollment were subsequently diagnosed with schizophrenia 6 months later. The inclusion criteria for patient group were age between 18 and 40, Han Chinese ethnicity, right-handedness, drug-naïve, first-episode. Exclusion criteria were inability to provide informed consent, substance abuse, mental retardation, significant medical conditions including severe cardiovascular, hepatic and renal diseases and pregnancy or breastfeeding, and a history of other forms of antipsychotic treatment. Detailed progress through phases of the trail was presented in Figure S1. Thirty two healthy controls were recruited from the local community via advertisement. Those with mental disorders, neurological diseases, or a positive family history of mental illness were excluded. This study was approved by the Institutional Review Board of the Shanghai Mental Health Center, and written informed consent was obtained from all participants.

For the 46 baseline patients and 32 controls enrolled in this study, 8 of them (7 patients and 1 controls) were excluded due to incomplete scans, and 8 of them (5 patients and 3 controls) were excluded due to excessive head movement. For the 30 patients completed a second MRI scanning at 8 weeks, 4 of them were excluded due to incomplete scans, and 2 of them were excluded due to excessive head movement. The dynamic functional connectivity and structural connectivity analyses were carried out in the remaining 34 patients at baseline, 24 patients at follow-up, and 28 controls.

The patients participated in the longitudinal study were treated with SGAs for 8 weeks. Drug choices and dosages were determined based on the treating psychiatrists’ clinical judgment. The details regarding medication and dosage for analyzed patient are provided in Table S1.

## Overview of the longitudinal connectivity analyses

A schematic diagram of the analysis framework to study the antipsychotic treatment-induced changes in structural connectivity and dynamic functional connectivity for first-episode schizophrenia is presented in Figure S2. Specifically, there are five analysis steps in this framework. First, a group independent component analysis (ICA) was performed to decompose the preprocessed fMRI data into multiple independent components (ICs), and the intrinsic connectivity networks (ICNs) among these ICs were identified according to their spatial activation maps. Second, to estimate dynamic connectivity patterns, we divided the time courses of each ICN into a set of windows by using a sliding window approach, and the covariance matrix within each window was subsequently calculated as functional brain networks. Third, a systematical dFC analysis (i.e. the temporal properties of FC states, dynamic topological property and temporal variability of FC) was implemented to investigate the dynamic network connectivity properties in schizophrenia. Forth, an SC association analysis was performed to investigate the SC counterparts of altered dFC and their couplings in pre-treatment patients. Finally, a longitudinal analysis was implemented to examine the effects of medications on the brain network measures and to find the associations between the brain changes and the improvement of psychotic symptoms. The detailed processing approaches of the above-mentioned steps were presented in the manuscript as well as in the following sections.

## Data acquisition

All participants were scanned on a 3.0 T Siemens Tim Verio scanner with a 32-channel head coil (Siemens, Erlangen, Germany) at Shanghai Mental Health Center. Prior to data acquisition, participants were instructed to stay awake with their eyes closed, to remain still, and not to think systematically during the scan. To minimize the head motion, participants’ heads were stabilized in the head coil using foam pads. High-resolution structural 3D T1-weighted images were acquired using a magnetization prepared rapid gradient-echo sequence (repetition time [TR] = 2530 ms; echo time [TE] = 3.65 ms; flip angle = 7°; slice thickness = 1 mm; slices = 224; acquisition matrix = 256 × 256; field of view [FOV] = 256 × 256 mm^2^; voxel size = 1 × 1 × 1 mm^3^). Resting-state fMRI scans were acquired using an echo-planar imaging sequence (TR = 2000 ms; TE = 30 ms; flip angle = 90°; slice thickness = 4 mm; slices = 30; in-plane matrix resolution = 64 × 64; FOV = 220 × 220 mm^2^; 180 volumes; and a total of 6 min). DWI scans were obtained using a 2D echoplanar diffusion sequence (number of diffusion encoding directions = 35; b-value = 1000 s/mm^2^; number of non-diffusion (b0) images = 6; number of repetitions = 2; TR = 10200 ms; TE = 90 ms; slice thickness = 2 mm; slices = 74; acquisition matrix = 128 × 128; FOV = 256 × 256 mm^2^; voxel size = 2 × 2 × 2 mm^3^).

Of note, we did not scan the healthy controls at follow-up, which would ensure the stability of our measures and thus contribute to clarify the association mechanism of observed changes in patients at 8 weeks. Similar approaches were also adopted in several recent longitudinal schizophrenia studies ^2,3^. However, other studies have suggested that this design may lead to a potential repetition effects ^4,5^. Two scans at similar time interval for healthy controls may improve potential repetition effects in future studies.

## Functional image preprocessing

Resting-state functional images were preprocessed using the DPARSF ^6^ based on SPM 12 (http://www.fil.ion.ucl.ac.uk/spm). The main preprocessing included four steps as follows. First, the 5 initial volumes were discarded to stabilize the signal and adapt to inherent scanner noise. Second, the remaining images were corrected by slice-timing calibrating and head motion realigning. Specifically, they were first corrected differences in within-scan acquisition time among slices and then were realigned to the first volume for the purpose of correcting inter-scan head motion. Next, the images were spatially normalized to a standard three-dimensional space with the Montreal Neurological Institute (MNI) template and resampled to a resolution of 3 × 3 × 3 mm^3^. Here, a rigid-body transformation was used to co-register the individuals’ the high resolution structural images to the mean functional images, and all the co-registered images were segmented into gray matter, white matter and cerebrospinal fluid in MNI space. Finally, the images were spatial smoothed using a Gaussian kernel of 6 mm full width at half maximum. Moreover, to account for artifact of head motion, any data affected by head motion (maximal motion between volumes in each direction is greater than 1.5 mm, and rotation in each axis is greater than 1.5°) were discarded. We also calculated the mean frame-wise displacement (FD) of each participant on the basis of realignment parameters and the participants with FD more than 0.2 mm were excluded ^7^. There was no significant group difference in FD between the remaining baseline patients and controls (SZ_b: 0.14 ± 0.0734; HC: 0.14 ± 0.0558; *P* = 0.79, two-sample t-test). The head motion was also corrected in the subsequent statistical comparisons through regarding mean FD as covariate.

## Diffusion image preprocessing

DWI images of each participant were preprocessed using the Pipeline for Analyzing Brain Diffusion Images (PANDA) ^8^. The main preprocessing included four steps as follows. First, brain mask was estimated by removing the skull from the b0 image. Second, the non-brain space in the raw images was removed to reduce the memory cost and speed up the processing in subsequently steps. The acquired brain mask was used to determine the borders of the brain along the three dimensions. Third, eddy-current induced distortion of diffusion-weighted images, as well as simple head motion during scanning, was corrected by registering the DWI images to the b0 image with an affine transformation. Finally, the diffusion tensor metrics including fractional anisotropy (FA), mean diffusivity, axial diffusivity and radial diffusivity maps were calculated.

## Group independent component analysis

In line with previous literature investigating dynamic functional network connectivity ^9,10^, we used a standard pipeline of spatial GICA to decompose preprocessed fMRI data into different independent components (ICs). Of note, we performed global signal regression (GSR) in the GICA framework and implemented by GIFT toolbox. Specifically, the global mean signal per time point was removed as the standard principal component analysis (PCA) processing step prior to ICA, in line with previous dFC studies ^11,12^. Subsequently, the PCA was conducted to reduce subject-specific data into 120 principal components. Next, subject-reduced data of all subjects across time were concatenated and further reduced into 100 ICs by using an infomax algorithm ^13^. Here, the infomax ICA algorithm was repeated 20 times in ICASSO (http://research.ics.tkk.fi/ica/icasso/) to ensure the reliability and stability of decomposition. After estimating the group spatial maps, a back reconstruction of subject-specific spatial maps and corresponding time courses was performed by using a group information-guided ICA approach. The intrinsic connectivity networks (ICNs) among the 100 ICs were identified according to the criteria as follows. First, peak activation coordinates were located primarily in gray matter. Second, low spatial overlap with known vascular, ventricular, motion, and susceptibility artifacts. Third, time courses were dominated by low-frequency fluctuations ^11,14^. We finally identified 36 ICNs among 100 ICs that wed grouped into 7 resting-state networks (RSNs), based on the spatial correlation values between ICs and the template ^11,15^. As shown in Figure S3, these RSNs were arranged into sub-cortical (SUC; 2 ICNs), auditory (AUD; 2 ICNs), visual (VIS; 5 ICNs), somatomotor (SM; 9 ICNs), cognitive control (CC; 9 ICNs), default mode (DM; 7 ICNs), and cerebellar (CB; 2 ICNs) networks. The activation information spatial maps of 36 ICNs are presented in Table S2. Additionally post-processing were further performed on the time courses of the identified ICNs to remove remaining noise sources, including detrending linear, quadratic and cubic trends, conducting regressions of the 6 realignment parameters and their temporal derivatives, despiking detected outliers, and low-pass filtering with a cutoff frequency of 0.15 Hz.

## Structural connectivity estimation

To assess the effects of treatment on structural underpinnings of altered dFC, we tracked the white matter fibers between pairs of ICNs that showed altered dFC variability in baseline patients. The ICNs in the MNI space were transformed into the native DWI space of each participant. First, the individual FA image in the native space was co-registered to its b0 image using a linear transformation. Then, the transformed b0 image was non-linearly registered to the ICBM152 template. Based on the resultant transformations in these two steps, an inverse warping transformation from the MNI space to the native DWI space can be obtained. We subsequently performed deterministic tractography in the native space for each participant using the fiber assessment by continuous tracking (FACT) algorithm ^16^. Here, fiber tracking was terminated when the angle between two consecutive orientations was >45° or when the FA value was <0.2. Given that the outcome of tractography is affected by the initial position of the seed points within the voxel ^17^, 100 seeds were randomly selected within each voxel to avoid biases from initial seed positioning. Resultant whole-brain tracts provided the edges for building the structural connectome. In line with previous studies ^2,18,19^, SC weight was defined as the number of deterministic streamlines connecting each pair of ICNs.

## Validation analysis

To verify the consistency of the results across different sliding window sizes, we estimated the dynamic functional network connectivity and tested group differences in other window sizes (18-26 TRs: 36-52 s). Since the number of clusters in the range of 4-6 were shown to be reasonable choices in previous dynamic studies, we also conducted the k-means clustering analysis using 4 and 6 as the number of clusters (*k*=4, 6).

# Supplementary Results and Discussion

## Group-specific cluster centroids for each state

Fig. S4 shows group-specific dFC patterns with the window size of 22 TRs and 5 as the number of clusters. We observed that in each group, State 3 was characterized by strongly positive couplings among SUC, AUD, SM, and CC, as well as negative DM couplings with SUC, AUD, SM, and CC, and State 5 showed stronger connections between sensory networks.

## Occurrences of dynamic functional connectivity patterns in different window sizes

To test the consistency of our results across sliding window sizes, we estimated the dynamic functional connectivity in other window sizes with 18 TRs and 26 TRs. We applied the k-means clustering analysis to cluster all windowed FC matrices into 5 different dynamic states. As shown in Fig. S5a and Fig. S6a, the cluster centroids of these FC states were similar to the dynamic patterns in the manuscript. We then explored the group difference in state occurrence and found that the baseline patients had a lower occurrence in State 5 than healthy controls (18 TRs: *P* = 0.0095, FDR-corrected; 26 TRs: *P* = 0.0097, FDR-corrected; see Fig. S5b and Fig. S6b), consistent with the results obtained by 22 TRs. To examine the effects of antipsychotic treatment on dynamic connectivity patterns, we further compared the treated patients to that paired baseline and observed a significant increase in the occurrence rate of State 3 for patients after treatment (18 TRs: *P* = 0.0089, FDR-corrected; 26 TRs: *P* = 0.0092, FDR-corrected; see Fig. S5c and Fig. S6c). In addition, a correlation analysis was implemented to investigate whether medication-induced changes in dynamic connectivity patterns can shape the relief of psychotic symptoms in patients. In line with the correlation analysis results in the manuscript, we found a significant association between the changes in occurrence rate of State 3 and the changes in PANSS scores for both 18 TRs (*r* = 0.42, *P* = 0.0410; Fig. S5d) and 26 TRs (*r* = 0.44, *P* = 0.0306; Fig. S6d). Taken all these results together, our findings revealing the occurrence of dynamic FC states and its association with relief of psychotic symptoms in first-episode schizophrenia were consistent using different lengths of sliding window.

## Occurrences of dynamic functional connectivity patterns in different cluster number

We also tested the consistency of the results across different number of clusters. We conducted the k-means clustering analysis at different number of clusters (i.e. k = 4 and 6). As shown in Fig. S7a and Fig. S8a, we found that the cluster centroids of these FC states were similar to the patterns as mentioned previously. To investigate the effects of antipsychotic treatment on dynamic connectivity patterns of schizophrenia, we first made a comparison between baseline patient and healthy control groups and found a significant group difference in state occurrence. Specifically, the baseline patients had a lower occurrence in State 4 at the condition of 4 clusters than controls (*P* = 0.0089, FDR-corrected; Fig. S7b) and had a lower occurrence in State 5 at the condition of 6 clusters than controls (*P* = 0.0095, FDR-corrected; Fig. S8b), and these group differences were consistent with the results in the manuscript. We then explored the longitudinal changes of state occurrence in response to antipsychotic treatment for schizophrenia patients. As shown in Fig. S7c and Fig. S8c we observed a significant increase in the occurrence rate of State 2 at the condition of 4 clusters (*P* = 0.0097, FDR-corrected) and in the occurrence rate of State 3 at the condition of 6 clusters (*P* = 0.0088, FDR-corrected), and these state shared similar connectivity patterns with the State 3 in the manuscript. We further calculated the correlation between the longitudinal changes of state occurrence and the longitudinal changes of clinical symptoms (Fig. S7d and Fig. S8d). For both 4 and 6 clusters, the increased state occurrence was positively correlated with the improvement of psychopathologic scores (4 clusters: *r* = 0.47, *P* = 0.0106; 6 clusters: *r* = 0.49, *P* = 0.0077), consistent with the results obtained by 5 clusters. Taken all these results together, our findings revealing the occurrence of dynamic FC states and its association with relief of psychotic symptoms in first-episode schizophrenia were consistent using different numbers of clusters.

## Occurrences of dynamic functional connectivity patterns without GSR

The global signal regression (GSR) remains controversial. Therefore, we have performed additional analyses without GSR and reanalyzed the dynamic FC reorganization; we found that the main findings were intact (see Fig. S9).

## Dynamic network efficiency

To assess parallel information transfer in functional networks, we calculated the dynamic network efficiency for each participants and compared it between groups. We did not find significant difference in dynamic network efficiency between baseline patient and control groups (Fig. S10). Previous studies have reported alterations in dynamic global efficiency for schizophrenia patients ^20,21^. The discrepancies could stem from different disease duration or treatment for patients enrolled in this study and previous work, that is a drug-naïve, first-episode patients cohort in this study in comparison with chronic or medicated patients in the works of Yu et al and Sun et al, suggesting a weaker influence on dynamic topological property in patients at the early phase of schizophrenia ^2^. We further investigated the longitudinal changes in dynamic network efficiency of schizophrenia before and after treatment. There was no significant difference in both global and local efficiency between patients at follow-up and those at baseline. Previous study has suggested that the network topological property in first-episode schizophrenia was not altered after a short-term antipsychotic treatment ^2^. Thus, it is possible that 8 weeks of antipsychotic treatment is too short a time period to observe the group difference in network efficiency. Intriguingly, we found that the changed dynamic global efficiency was significantly correlated with the decreased PANSS scores (*r* = 0.50, *P* = 0.0138; Fig. S10b), suggesting that the antipsychotic drugs might attenuate symptoms by modulating global efficiency of the brain networks. In addition, we found a significant anti-correlation between global efficiency and psychopathologic score in patients after treatment, but did not find any significant correlations at baseline. This relationship established after treatment could stem from the heterogeneity of treatment outcomes in schizophrenia, and perhaps some patients are more sensitive to treatment, resulting in more increased network efficiency after treatment ^22^.

# Supplementary Tables and Figures

## Table S1. The information of medication and dosage for each patient.

| **No.** | **Gender** | **Age** | **Medication** | **Dosage**^*^  **(mg)** |
| --- | --- | --- | --- | --- |
|  |  |  |  |  |
| 1 | M | 19 | Paliperidone | 200 |
| 2 | F | 30 | Clozapine | 500 |
| 3 | M | 34 | Risperidone | 300 |
| 4 | F | 23 | Risperidone | 200 |
| 5 | F | 33 | Paliperidone | 200 |
| 6 | F | 21 | Olanzapine | 400 |
| 7 | M | 32 | Paliperidone | 200 |
| 8 | F | 26 | Olanzapine | 400 |
| 9 | F | 25 | Paliperidone | 200 |
| 10 | F | 28 | Risperidone | 200 |
| 11 | M | 25 | Olanzapine | 400 |
| 12 | M | 31 | Risperidone | 300 |
| 13 | F | 27 | Olanzapine | 400 |
| 14 | F | 24 | Aripiprazole | 400 |
| 15 | M | 21 | Paliperidone | 150 |
| 16 | M | 29 | Olanzapine | 400 |
| 17 | M | 40 | Olanzapine | 400 |
| 18 | M | 29 | Olanzapine | 300 |
| 19 | F | 19 | Olanzapine | 400 |
| 20 | M | 24 | Paliperidone | 300 |
| 21 | M | 30 | Olanzapine | 300 |
| 22 | F | 24 | Aripiprazole | 400 |
| 23 | F | 40 | Quetiapine | 800 |
| 24 | M | 25 | Olanzapine | 400 |

^*^ The daily antipsychotic dose was converted to chlorpromazine equivalents (CPZE).

## Table S2. Peak activation information of the 36 ICNs

| **ICNs** | **Anatomical regions** | **AAL** | **BA** | **MNI coordinates^*^** | | | **Cluster size** | ***T*-value** |
| --- | --- | --- | --- | --- | --- | --- | --- | --- |
|  |  |  |  | X | Y | Z |  |  |
| **SUC (2)** |  |  |  |  |  |  |  |  |
| ■ IC2 | L Putame | Putamen_L | - | -27 | -3 | -3 | 404 | 37.93 |
|  | R Putame | Putamen_R | - | 27 | -3 | 0 | 406 | 34.68 |
| ■ IC18 | L Thalamus | Thalamus_L | - | -15 | -18 | 0 | 376 | 26.79 |
|  | R Thalamus | Thalamus_R | - | 15 | -15 | 3 | 369 | 24.35 |
| **AUD (2)** |  |  |  |  |  |  |  |  |
| ■ IC38 | R superior temporal gyrus | Temporal_Sup_R | 22 | 54 | -33 | 3 | 745 | 29.36 |
| ■ IC96 | L superior temporal gyrus | Temporal_Sup_L | 22 | -60 | -24 | 9 | 523 | 32.66 |
|  | R superior temporal gyrus | Temporal_Sup_R | 22 | 60 | -6 | 0 | 523 | 28.91 |
| **VIS (5)** |  |  |  |  |  |  |  |  |
| ■ IC14 | B cuneus | Calcarine_L & _R | 18 | 3 | -81 | 9 | 974 | 32.75 |
| ■ IC60 | L fusiform gyrus | Fusiform_L | 37 | -30 | -45 | -18 | 389 | 26.03 |
|  | R fusiform gyrus | Fusiform_R | 37 | 30 | -42 | -18 | 357 | 27.10 |
| ■ IC66 | L middle temporal gyrus | Temporal_Mid_L | 39 | -45 | -72 | 12 | 254 | 22.49 |
|  | R middle temporal gyrus | Temporal_Mid_R | 39 | 48 | -57 | 9 | 492 | 23.70 |
| ■ IC78 | L middle temporal gyrus | Temporal_Mid_L | 22 | -57 | -45 | 6 | 689 | 24.66 |
| ■ IC90 | B cuneus | Cuneus_L & _R | 19 | 0 | -81 | 21 | 977 | 28.78 |
| **SM (9)** |  |  |  |  |  |  |  |  |
| ■ IC5 | L postcentral gyrus | Postcentral_L |  |  |  |  |  |  |
|  | R postcentral gyrus | Postcentral_R | 6 | -48 | -12 | 39 | 473 | 30.06 |
| ■ IC16 | L postcentral gyrus | Postcentral_L | 3 | -39 | -27 | 57 | 850 | 26.69 |
| ■ IC17 | B precentral gyrus | Paracentral_Lobule_L & _R | 4 | -9 | -33 | 69 | 1003 | 28.90 |
| ■ IC19 | R precentral gyrus | Precentral_R | 4 | 39 | -21 | 57 | 840 | 27.27 |
| ■ IC27 | L postcentral gyrus | Postcentral_L | 7 | -18 | -54 | 69 | 521 | 24.42 |
|  | R postcentral gyrus | Postcentral_R | 7 | 21 | -51 | 69 | 486 | 25.27 |
| ■ IC32 | B supplementary motor area | Supp_Motor_Area_L & _R | 6 | 18 | -9 | 60 | 878 | 24.57 |
| ■ IC33 | L precentral gyrus | Precentral_L | 6 | -45 | -6 | 51 | 360 | 20.25 |
|  | R precentral gyrus | Precentral_R | 6 | 48 | 0 | 48 | 302 | 19.49 |
| ■ IC46 | B cingulate gyrus | Cingulum_Mid_L & _R | 24 | 3 | 3 | 45 | 792 | 26.77 |
| ■ IC54 | L postcentral gyrus | Postcentral_L | 1 | -57 | -27 | 39 | 539 | 28.56 |
|  | R postcentral gyrus | Postcentral_R | 3 | 57 | -21 | 39 | 232 | 21.41 |
| **CC (9)** |  |  |  |  |  |  |  |  |
| ■ IC12 | R inferior frontal gyrus | Frontal_Inf_Tri_R | 45 | 63 | 21 | 21 | 542 | 25.00 |
| ■ IC20 | L insula | Insula_L | 13 | -42 | 0 | -3 | 410 | 26.31 |
|  | R insula | Insula_R | 13 | 39 | 12 | -15 | 377 | 29.10 |
| ■ IC45 | L inferior parietal lobule | Parietal_Inf_L | 40 | -42 | -42 | 45 | 468 | 23.98 |
|  | R inferior parietal lobule | Parietal_Inf_R | 40 | 33 | -51 | 51 | 529 | 24.45 |
| ■ IC53 | L inferior frontal gyrus | Frontal_Inf_Tri_L | 46 | -45 | 36 | 15 | 302 | 24.97 |
|  | R inferior frontal gyrus | Frontal_Inf_Tri_R | 46 | 45 | 39 | 15 | 408 | 25.58 |
| ■ IC59 | B supplementary motor area | Supp_Motor_Area_L & _R | 6 | -3 | 3 | 63 | 694 | 26.74 |
| ■ IC81 | L middle frontal gyrus | Frontal_Mid_L | 9 | -27 | 54 | 33 | 563 | 28.81 |
|  | R middle frontal gyrus | Frontal_Mid_R | 9 | 36 | 51 | 30 | 267 | 21.18 |
| ■ IC82 | L inferior parietal lobule | Parietal_Inf_L | 40 | -54 | -42 | 42 | 353 | 27.07 |
|  | R inferior parietal lobule | Parietal_Inf_R | 40 | 60 | -42 | 36 | 397 | 23.71 |
| ■ IC86 | R superior frontal gyrus | Frontal_Sup_R | 10 | 27 | 60 | 18 | 515 | 22.80 |
| ■ IC94 | L superior frontal gyrus | Frontal_Sup_L | 8 | -21 | 24 | 54 | 431 | 23.67 |
|  | R superior frontal gyrus | Frontal_Sup_R | 8 | 27 | 30 | 45 | 401 | 22.43 |
| **DM (7)** |  |  |  |  |  |  |  |  |
| ■ IC37 | B precuneus | Precuneus_L & _R | 7 | 6 | -66 | 57 | 986 | 34.17 |
| ■ IC51 | L anterior cingulate cortex | Cingulum_Ant_L & _R | 32 | 3 | 39 | 18 | 896 | 30.93 |
| ■ IC64 | B precuneus | Precuneus_L & _R | 7 | 9 | -66 | 30 | 732 | 29.72 |
| ■ IC69 | L angular gyrus | Angular_L | 40 | -45 | -66 | 45 | 512 | 30.03 |
| ■ IC77 | B posterior cingulate cortex | Cingulum_Post_L & _R | 31 | -6 | -57 | 18 | 573 | 27.17 |
| ■ IC85 | R angular gyrus | Angular_R | 40 | 45 | -57 | 33 | 576 | 28.38 |
| ■ IC97 | B medial frontal gyrus | Frontal_Sup_Medial_L & _R | 9 | 0 | 57 | 15 | 904 | 31.15 |
| **CB (2)** |  |  |  |  |  |  |  |  |
| ■ IC22 | B Cerebellum | Cerebelum_6_L & _R | - | -27 | -60 | -39 | 936 | 25.17 |
| ■ IC42 | B Cerebellum | Cerebelum_6_L & _R | - | -24 | -69 | -21 | 950 | 31.85 |

^*^The coordinates are peak voxel coordinates of the one-sample t-test results for each component spatial maps of all participants. A color-coded legend of each IC number matches to the overlaid colors of the spatial maps in Figure S2. Abbreviations: ICNs, intrinsic connectivity networks; IC, independent component; BA, Brodmann area; L, left; R, right; B, bilateral.

Screened

(n=198)

Consented

(n=59)

Completed 8-week study

(n=24)

Entered into antipsychotic trail

(n=46)

Excluded (n=139)

- Not meeting diagnostic criteria (n=38)
- Not interesting in participation (n=42)
- Medical condition (n=31)
- Agitation (n=15)
- Others (n=13)

Drop before start of antipsychotic trail (n=13)

- Withdrew consent (n=4)
- Did not tolerate scan environment (n=5)
- lost to follow-up(n=4)

Discontinued treatment (n=10)

- Excessive head movement (n=2)
- Incomplete scans(n=4)
- Withdrew consent (n=2)
- lost to follow-up(n=2)

Baseline study

(n=34)

Discontinued treatment (n=12)

- Excessive head movement (n=5)
- Incomplete scans(n=7)

## Fig. S1 Flow chart showing the inclusion of patients for this longitudinal study.


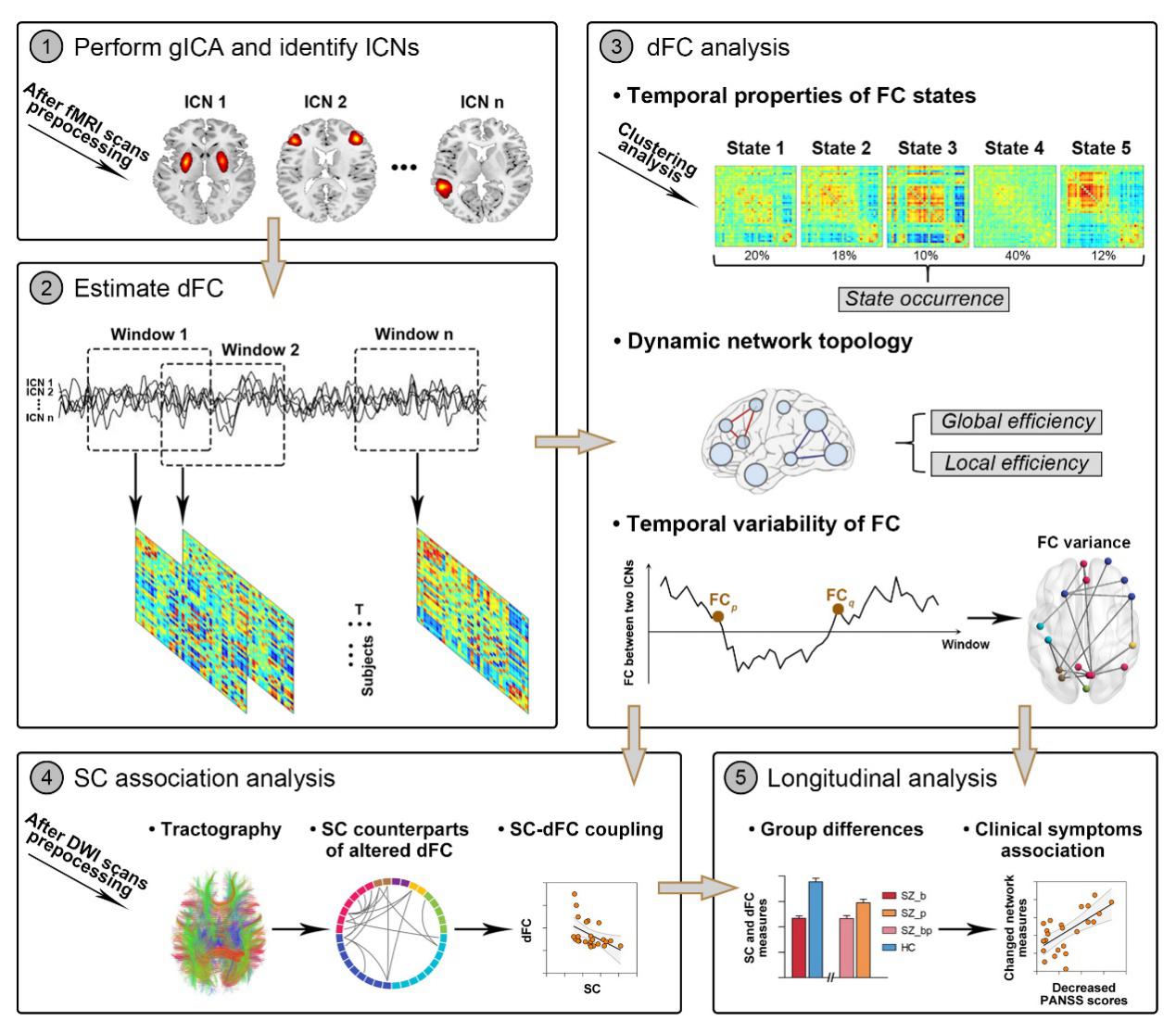


## Fig. S2 Analysis flowchart to study brain network connectivity in first-episode schizophrenia.


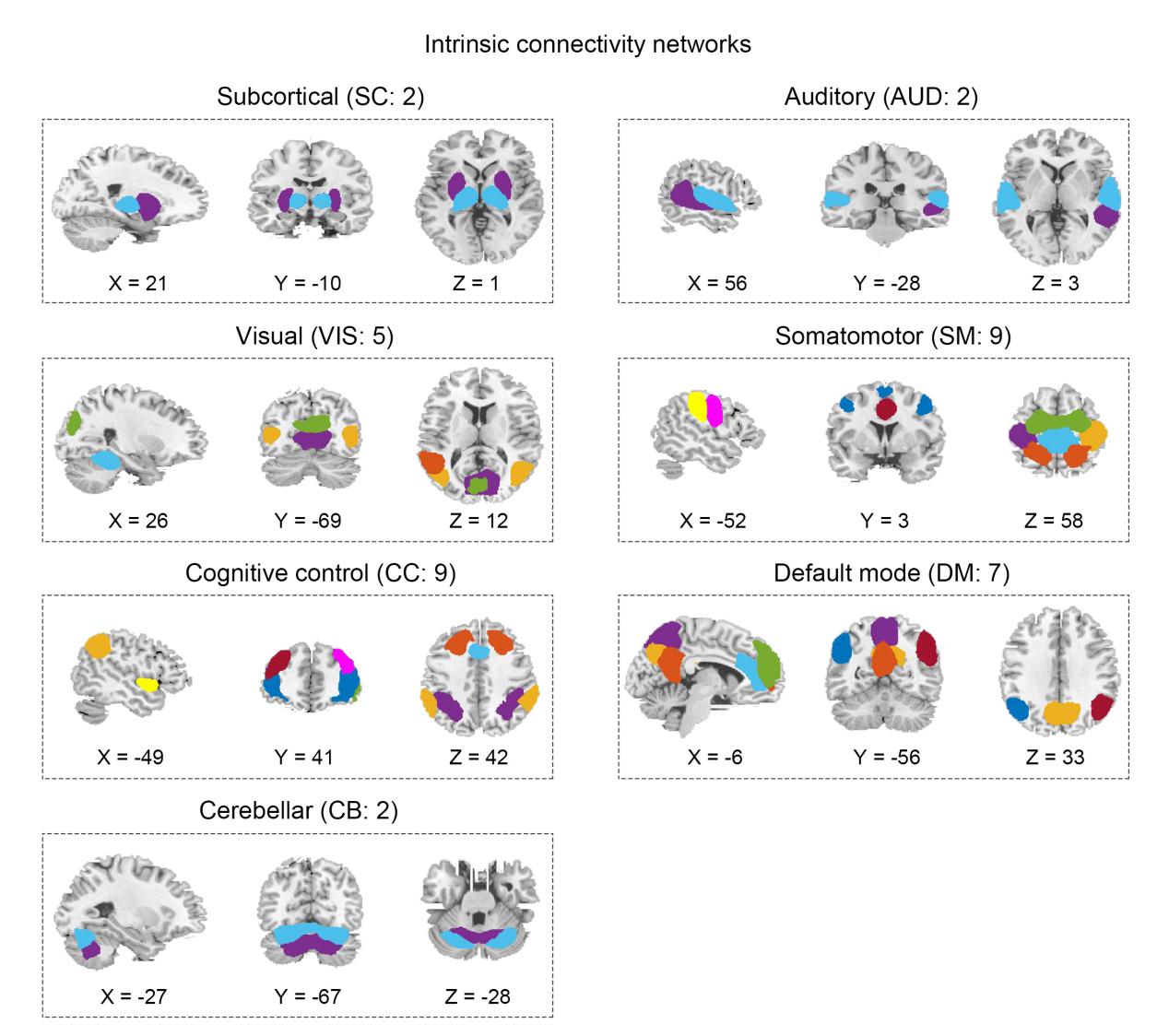


Fig. S3 Composite maps of the 36 identified ICNs. These ICNs were sorted into seven subcategories. Each color in the composite maps corresponds to a different ICN.


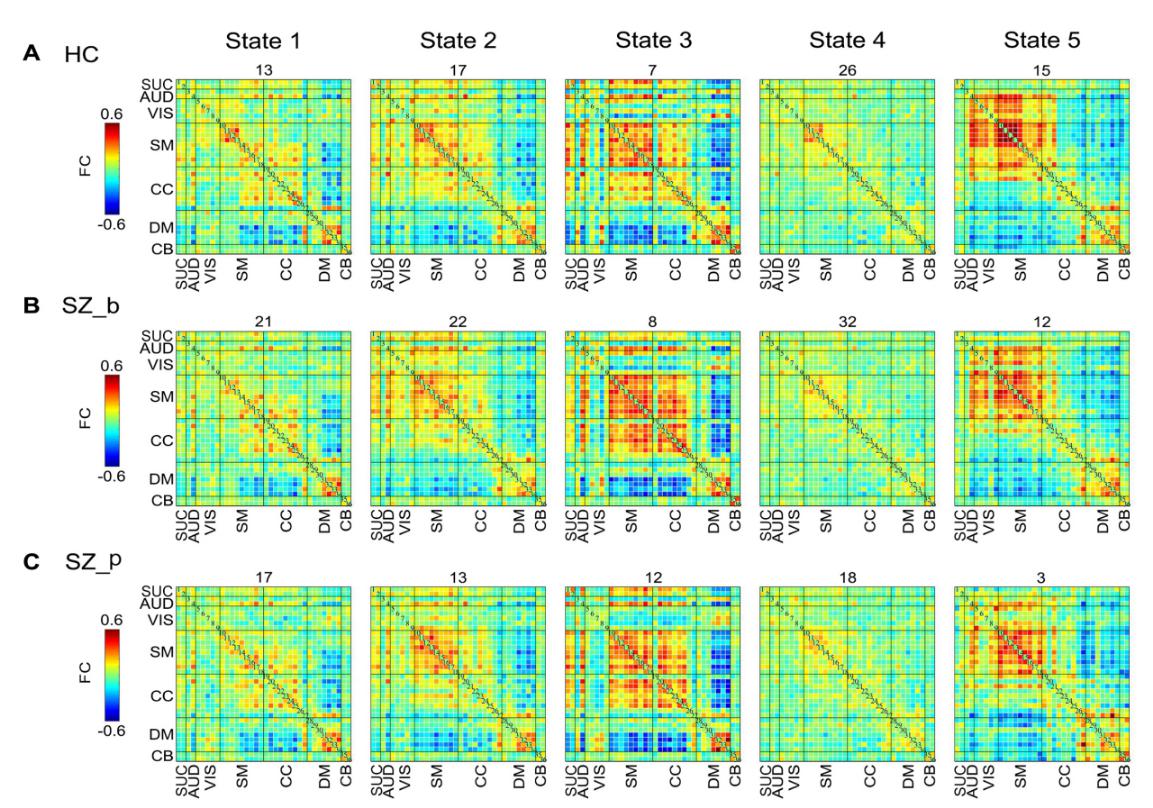


Fig. S4 Group-specific centroid matrices. Centroid matrices of each state for healthy control (a), baseline patient (b) and follow-up patient (c) groups. The number listed above each cluster centroid is the count of participants that had at least one window in this state.


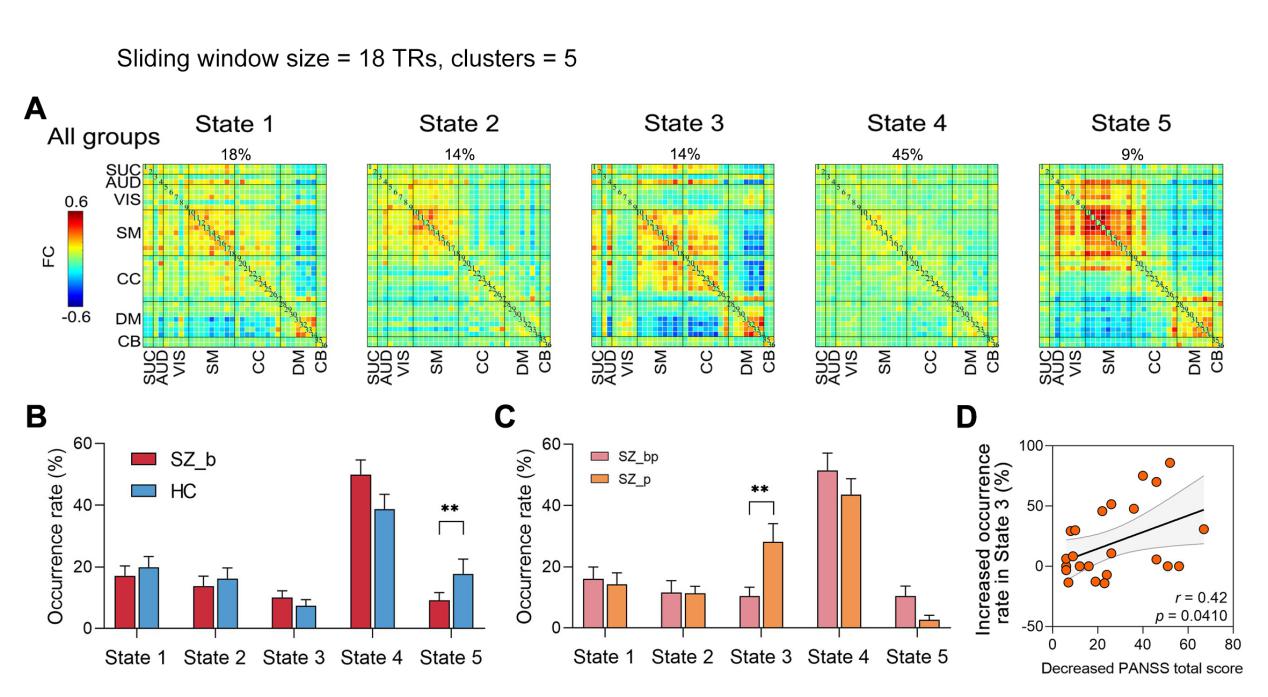


Fig. S5 Dynamic functional connectivity patterns with 18 TRs. (a) Five discrete dynamic functional connectivity patterns across all groups. The percentage of occurrences is listed above each cluster centroid. The color bar represents z value of functional connectivity (FC). (b) Differences in state occurrences between baseline schizophrenia patient (SZ_b) and healthy control (HC) groups. (c) Group differences in state occurrences between post-treatment (SZ_p) and paired baseline patients (SZ_bp). (d) Associations between changed occurrences of the State 3 and improved psychosis symptoms after treatment. ***P*<0.01, FDR-corrected.

**
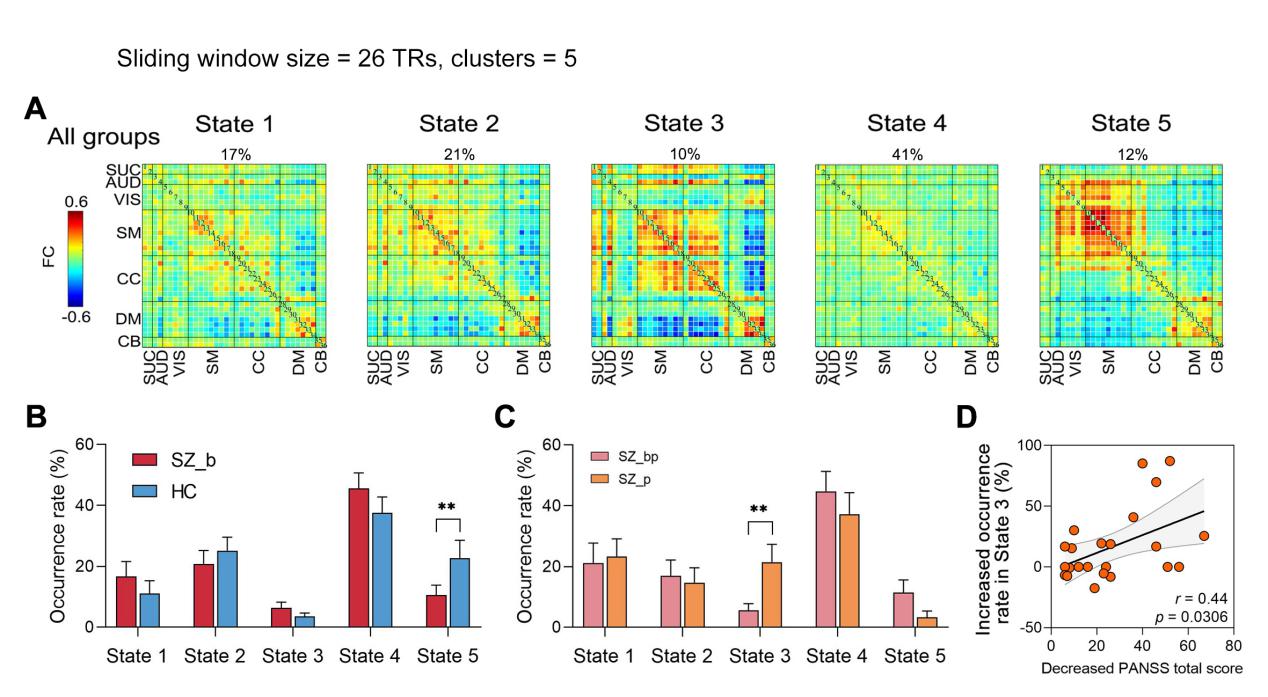
**

Fig. S6 Dynamic functional connectivity patterns with 26 TRs. (a) Five discrete dynamic functional connectivity patterns across all groups. The percentage of occurrences is listed above each cluster centroid. The color bar represents z value of functional connectivity (FC). (b) Differences in state occurrences between baseline schizophrenia patient (SZ_b) and healthy control (HC) groups. (c) Group differences in state occurrences between post-treatment (SZ_p) and paired baseline patients (SZ_bp). (d) Associations between changed occurrences of the State 3 and improved psychosis symptoms after treatment. ***P*<0.01, FDR-corrected.

**
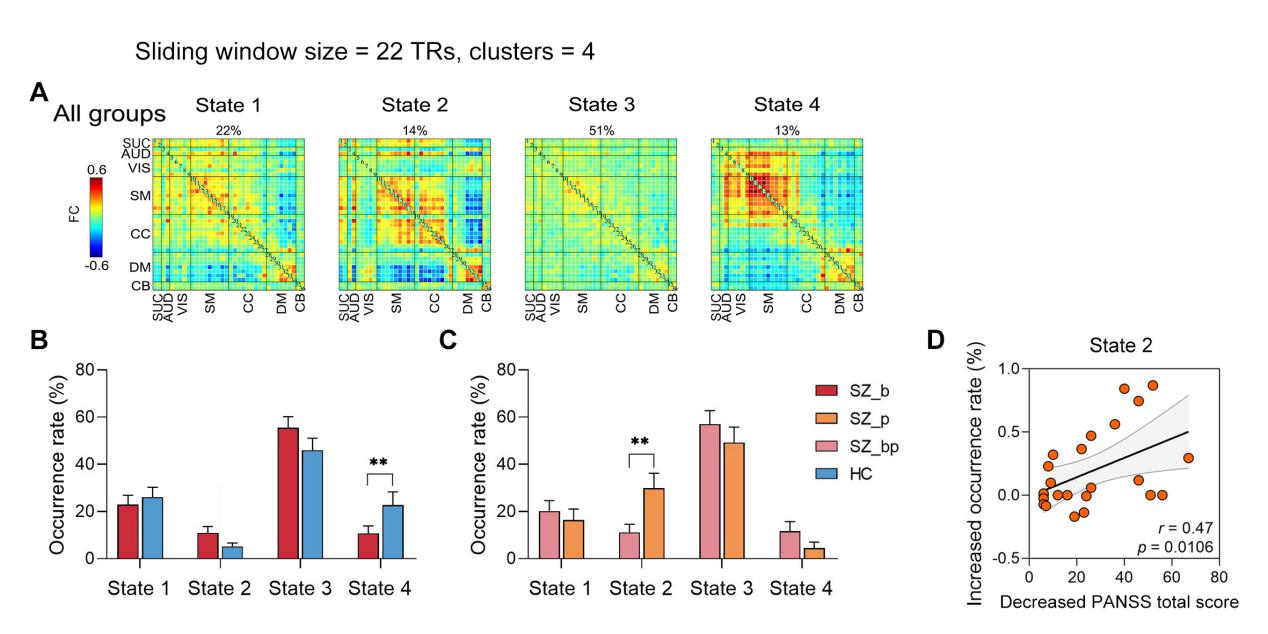
**

Fig. S7 Dynamic functional connectivity patterns with 4 clusters. (a) Four discrete dynamic functional connectivity patterns across all groups. The percentage of occurrences is listed above each cluster centroid. The color bar represents z value of functional connectivity (FC). (b) Differences in state occurrences between baseline schizophrenia patient (SZ_b) and healthy control (HC) groups. (c) Group differences in state occurrences between post-treatment (SZ_p) and paired baseline patients (SZ_bp). (d) Associations between changed occurrences of the State 2 and improved psychosis symptoms after treatment. ***P*<0.01, FDR-corrected.

**
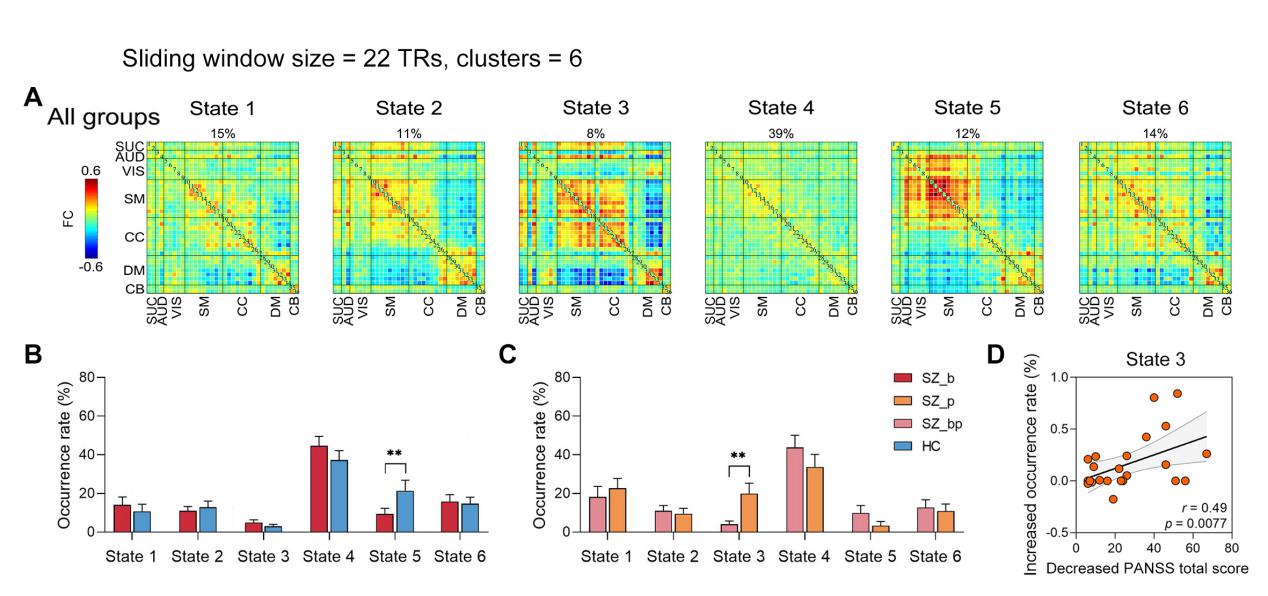
**

Fig. S8 Dynamic functional connectivity patterns with 6 clusters. (a) Six discrete dynamic functional connectivity patterns across all groups. The percentage of occurrences is listed above each cluster centroid. The color bar represents z value of functional connectivity (FC). (b) Differences in state occurrences between baseline schizophrenia patient (SZ_b) and healthy control (HC) groups. (c) Group differences in state occurrences between post-treatment (SZ_p) and paired baseline patients (SZ_bp). (d) Associations between changed occurrences of the State 3 and improved psychosis symptoms after treatment. ***P*<0.01, FDR-corrected.


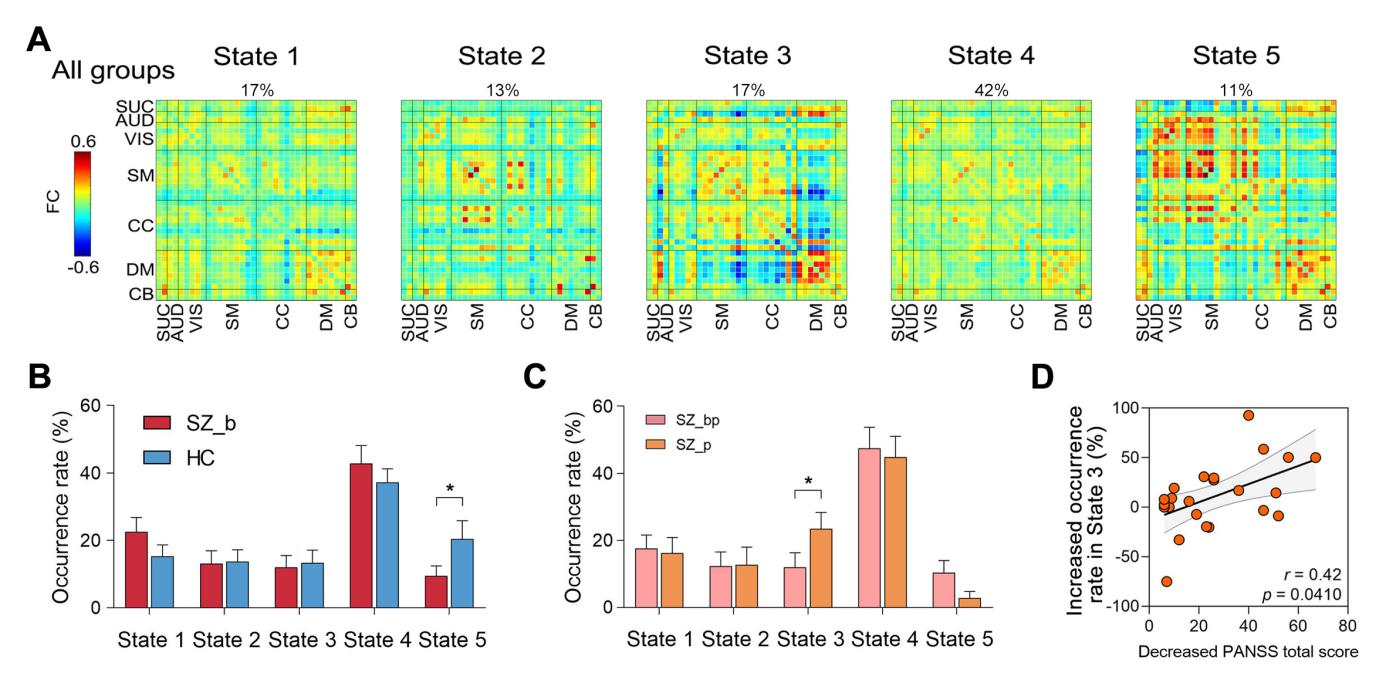


Fig. S9 Dynamic functional connectivity patterns without global signal regression. (a) Five discrete dynamic functional connectivity patterns across all groups. The percentage of occurrences is listed above each cluster centroid. The color bar represents z value of functional connectivity (FC). (b) Differences in state occurrences between baseline schizophrenia patient (SZ_b) and healthy control (HC) groups. (c) Group differences in state occurrences between post-treatment (SZ_p) and paired baseline patients (SZ_bp). (d) Associations between changed occurrences of the State 3 and improved psychosis symptoms after treatment. *P<0.05, FDR-corrected.


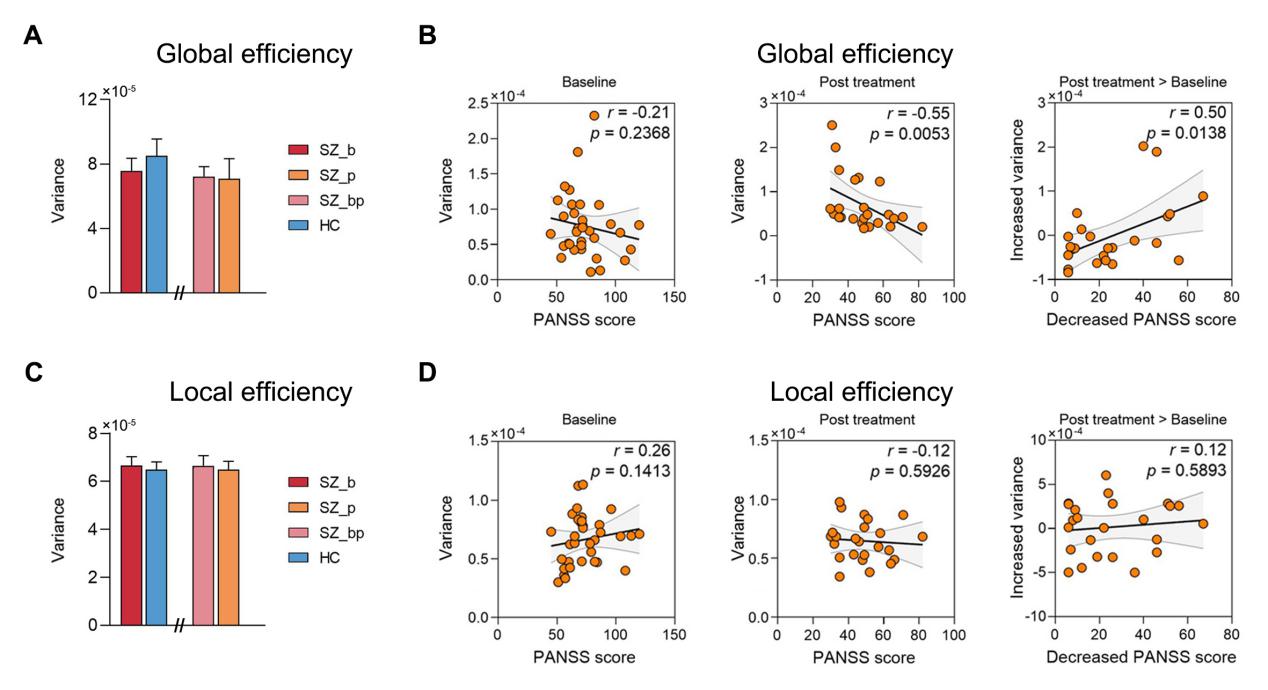


Fig. S10 Dynamic network efficiency and its association with clinical symptoms. The group differences in dynamic global efficiency (a) and local efficiency (c). The association between PANSS total score and dynamic global efficiency (b), as well as between PANSS total score and dynamic local efficiency (d).

**
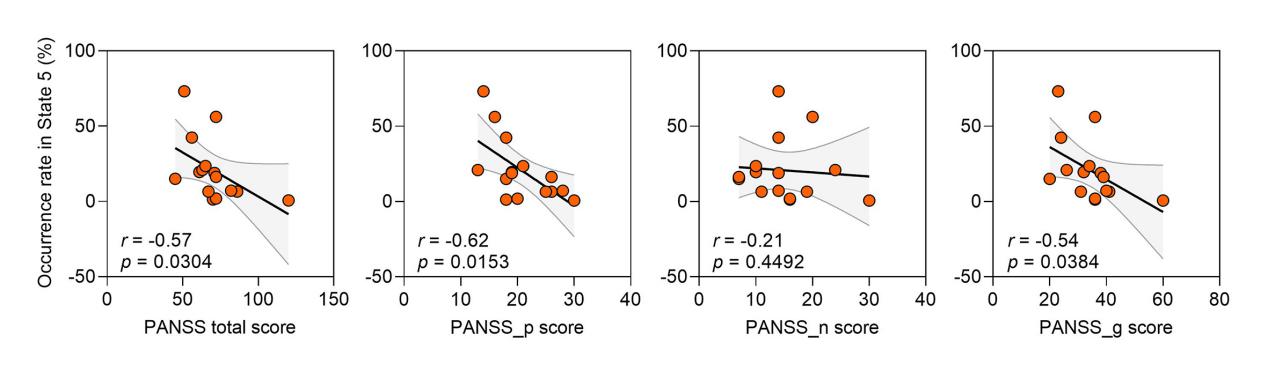
**

Fig. S11 Associations between the occurrence of State 5 and PANSS scores in baseline patients. The occurrence rate in State 5 was negatively correlated with the PANSS total score, positive symptoms (PANSS_p) score and general symptoms (PANSS_g) score. The correlation analysis was only performed for patients who had windows in this state. The statistical significance was *P*<0.05, with FDR-corrected.

**
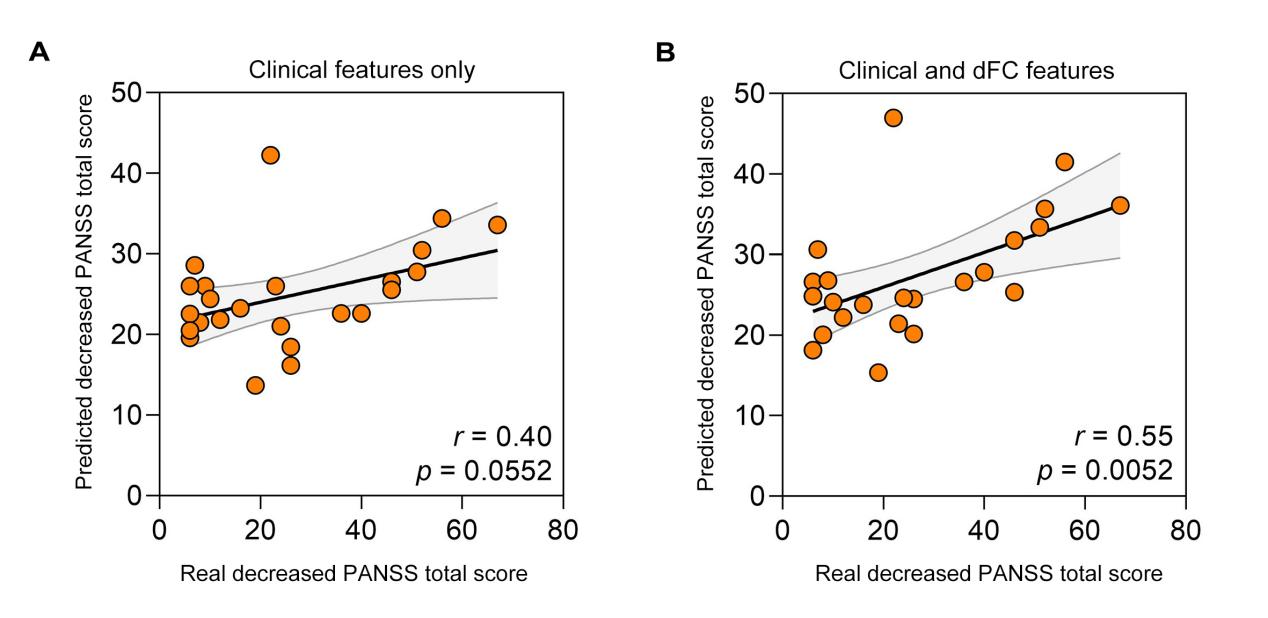
**

Fig. S12 Prediction of symptom reduction using clinical and brain network measure features. The decreased PANSS total score can be not predicted by clinical features only (a) but can be significantly predicted when combining with dynamic network connectivity features (b). The statistical significance was P<0.05, with FDR-corrected.

# Supplementary References

1 Sheehan, D. V. *et al.* The Mini-International Neuropsychiatric Interview (M.I.N.I.): the development and validation of a structured diagnostic psychiatric interview for DSM-IV and ICD-10. *The Journal of clinical psychiatry* **59 Suppl 20**, 22-33 (1998).

2 Crossley, N. A. *et al.* Connectomic correlates of response to treatment in first-episode psychosis. *Brain : a journal of neurology* **140**, 487-496, doi:10.1093/brain/aww297 (2017).

3 Duan, X. *et al.* Effect of Risperidone Monotherapy on Dynamic Functional Connectivity of Insular Subdivisions in Treatment-Naive, First-Episode Schizophrenia. *Schizophr Bull* **46**, 650-660, doi:10.1093/schbul/sbz087 (2020).

4 Murray, S. O., Olman, C. A. & Kersten, D. Spatially specific FMRI repetition effects in human visual cortex. *J Neurophysiol* **95**, 2439-2445, doi:10.1152/jn.01236.2005 (2006).

5 Tozzi, L., Goldstein-Piekarski, A. N., Korgaonkar, M. S. & Williams, L. M. Connectivity of the Cognitive Control Network During Response Inhibition as a Predictive and Response Biomarker in Major Depression: Evidence From a Randomized Clinical Trial. *Biological psychiatry* **87**, 462-472, doi:10.1016/j.biopsych.2019.08.005 (2020).

6 Yan, C. & Zang, Y. DPARSF: A MATLAB Toolbox for "Pipeline" Data Analysis of Resting-State fMRI. *Frontiers in systems neuroscience* **4**, 13, doi:10.3389/fnsys.2010.00013 (2010).

7 Power, J. D., Schlaggar, B. L., Lessov-Schlaggar, C. N. & Petersen, S. E. Evidence for hubs in human functional brain networks. *Neuron* **79**, 798-813, doi:10.1016/j.neuron.2013.07.035 (2013).

8 Cui, Z., Zhong, S., Xu, P., He, Y. & Gong, G. PANDA: a pipeline toolbox for analyzing brain diffusion images. *Frontiers in human neuroscience* **7**, 42, doi:10.3389/fnhum.2013.00042 (2013).

9 Tu, Y. *et al.* Abnormal thalamocortical network dynamics in migraine. *Neurology* **92**, e2706-e2716, doi:10.1212/WNL.0000000000007607 (2019).

10 Kim, J. *et al.* Abnormal intrinsic brain functional network dynamics in Parkinson's disease. *Brain : a journal of neurology* **140**, 2955-2967, doi:10.1093/brain/awx233/4320219 (2017).

11 Allen, E. A. *et al.* Tracking whole-brain connectivity dynamics in the resting state. *Cerebral cortex* **24**, 663-676, doi:10.1093/cercor/bhs352 (2014).

12 Tu, Y. *et al.* Distinct thalamocortical network dynamics are associated with the pathophysiology of chronic low back pain. *Nature communications* **11**, 3948, doi:10.1038/s41467-020-17788-z (2020).

13 Bell, A. J. & Sejnowski, T. J. An information-maximization approach to blind separation and blind deconvolution. *Neural Computing* **7**, 1129–1159 (1995).

14 Fiorenzato, E. *et al.* Dynamic functional connectivity changes associated with dementia in Parkinson's disease. *Brain : a journal of neurology* **142**, 2860-2872, doi:10.1093/brain/awz192 (2019).

15 Shirer, W. R., Ryali, S., Rykhlevskaia, E., Menon, V. & Greicius, M. D. Decoding subject-driven cognitive states with whole-brain connectivity patterns. *Cerebral cortex* **22**, 158-165, doi:10.1093/cercor/bhr099 (2012).

16 Mori, S., Crain, B. J., Chacko, V. P. & Van Zijl, P. C. Three-dimensional tracking of axonal projections in the brain by magnetic resonance imaging. . *Annals of neurology* **45**, 265–269 (1999).

17 Cheng, H. *et al.* Optimization of seed density in DTI tractography for structural networks. *J Neurosci Methods* **203**, 264-272, doi:10.1016/j.jneumeth.2011.09.021 (2012).

18 van den Heuvel, M. P. *et al.* Abnormal rich club organization and functional brain dynamics in schizophrenia. *JAMA psychiatry* **70**, 783-792, doi:10.1001/jamapsychiatry.2013.1328 (2013).

19 van den Heuvel, M. P., Scholtens, L. H., de Reus, M. A. & Kahn, R. S. Associated Microscale Spine Density and Macroscale Connectivity Disruptions in Schizophrenia. *Biological psychiatry* **80**, 293-301, doi:10.1016/j.biopsych.2015.10.005 (2016).

20 Yu, Q. *et al.* Assessing dynamic brain graphs of time-varying connectivity in fMRI data: application to healthy controls and patients with schizophrenia. *NeuroImage* **107**, 345-355, doi:10.1016/j.neuroimage.2014.12.020 (2015).

21 Sun, Y., Collinson, S. L., Suckling, J. & Sim, K. Dynamic Reorganization of Functional Connectivity Reveals Abnormal Temporal Efficiency in Schizophrenia. *Schizophr Bull* **45**, 659-669, doi:10.1093/schbul/sby077 (2019).

22 Carbon, M. & Correll, C. U. Clinical predictors of therapeutic response to antipsychotics in schizophrenia. *Dialogues Clin Neurosci* **16**, 505-524 (2014).
